# Gut microbiota alterations may increase the risk of prescription opioid use, but not vice versa: A two-sample bi-directional Mendelian randomization study

**DOI:** 10.3389/fmicb.2022.994170

**Published:** 2022-11-22

**Authors:** Liling Lin, Jianwei Lin, Junxiong Qiu, Feng Wei, Xiaohui Bai, Weiying Ma, Jingxian Zeng, Daowei Lin

**Affiliations:** ^1^Department of Anesthesiology, Sun Yat-sen Memorial Hospital, Sun Yat-sen University, Guangzhou, China; ^2^Big Data Laboratory, Joint Shantou International Eye Center of Shantou University and The Chinese University of Hong Kong, Shantou, Guangdong, China; ^3^Department of Cardiovascular Surgery, Sun Yat-sen Memorial Hospital, Sun Yat-sen University, Guangzhou, China

**Keywords:** causality, prescription opioid use, multisite chronic pain, gut microbiota, metabolites, Mendelian randomization

## Abstract

**Introduction:**

Gut microbiota alterations are strongly associated with prescription opioid use (POU) and multisite chronic pain (MCP). However, whether or not these associations are causal remains unknown. Therefore, we aim to explore the causal relationships between them comprehensively.

**Methods:**

A two-sample bi-directional Mendelian randomization was conducted to assess the potential associations between gut microbiota and POU/MCP using summary level Genome-wide association studies (GWASs) that were based on predominantly European ancestry.

**Results:**

Potential causal effects were identified between seven host genetic-driven traits of gut microbiota on POU, including *Adlercreutzia*, *Allisonella*, *Dialister*, *Anaerofilum*, *Anaerostipes*, *ChristensenellaceaeR.7group*, and *LachnospiraceaeNC2004group* at the genus level (*p* < 0.05) by the Inverse-variance weighted method, with significant causal effects of ChristensenellaceaeR.7group and *Allisonella* on POU (*p* < 0.025). A total of five genetically greater abundance of gut microbiota traits were identified to be possibly related to the level of MCP (*p* < 0.05), including genus *ErysipelotrichaceaeUCG003*, *family Clostridiaceae1*, order *Gastranaerophilales*, order *Actinomycetales*, and family *Actinomycetaceae*. In the other direction, no clear evidence was found to support a significant causal relationship between POU and gut microbiota, as well as MCP and gut microbiota. In addition, evidence was also provided for the relationship between triacylglycerols and diacylglycerol elevation, and an increased risk of POU and MCP. No evidence was found across various sensitivity analyses, including reverse causality, pleiotropy, and heterogeneity.

**Conclusion:**

The findings from this study provide robust evidence that gut microbiota alterations may be a risk of POU/MCP, but not vice versa.

## Introduction

Over the past two decades, the important role of gut microbiota has been recognized in the establishment and maintenance of health, as well as in the occurrence and progression of various diseases. A well-maintained gut microbiota diversity is essential for a normal life, whereas alterations to it (dysbiosis) have impacts on the gut-brain axis, leading to a variety of neurological diseases, such as neuropsychiatric disorders (Ochoa-Repáraz et al., 2020; Ni et al., 2021). Gut microbiota alterations are also noticed in patients suffering from different types and areas of chronic pain, including visceral pain, inflammatory pain, headache, neuropathic pain, and chronic widespread pain (Newlove-Delgado et al., 2019; Yang et al., 2019; Chen et al., 2020; Guida et al., 2020; Freidin et al., 2021). Based on these studies, we believe that two possibilities should not be neglected: one is that chronic pain may lead to dysbiosis, and the other is that gut microbiota may be a possible way to regulate chronic pain conditions.

Opioids are a major component in the treatment of chronic pain. While they do relieve pain, the long-term use of opioids is often accompanied by multiple systems disorders in the whole body, including the nervous, cardiovascular, digestive, immune, and endocrine systems (Farmer et al., 2018; de Vries et al., 2020; Hadland et al., 2021; Rosoff et al., 2021; Cai et al., 2022). Due to the side effects mentioned above, it has long been a public concern to reduce opioid use (Sandhu et al., 2018). Recent studies also demonstrated that the long-term use of opioid use may result in increasing comorbidity and behavioral changes due to dysbiosis (Meng et al., 2013, 2015; Wang et al., 2018). Furthermore, opioid receptors are found in both the digestive tract and the central nervous system, which suggests their important role in the modulation of the gut-brain axis (Zhang and Roy, 2021). Notably, observational animal studies also indicate the importance of the gut microbiota in opioid tolerance (Kang et al., 2017; Lee et al., 2018; Zhang et al., 2019).

Looking at the importance of gut microbiota in the gut-brain axis and the critical role of opioids in pain management, it is, therefore, crucial to reveal the causal direction between gut microbiota alteration and prescription opioid use (POU) as well as multisite chronic pain (MCP). A clear causal direction not only guides us on how to maintain a healthy gut microbiota composition but also provides us with a possible strategy for reducing opioid use. Conventionally, well-designed randomized controlled trials are the golden standard for inferring a causal relationship between gut microbiota and POU/MCP; however, they are difficult to implement due to ethical and legal restrictions. Mendelian randomization (MR) is an alternative approach to assess the causal relationship between exposures and outcomes, using genetic variants as unconfounded proxies for exposures (Emdin et al., 2017). Since genetic variants are randomly distributed during meiosis yielding, the MR approach is conceptually similar to a randomized controlled study and can minimize confounding such as social and economic factors (Emdin et al., 2017). Based on the advantages of MR mentioned above, we applied a two-sample bi-directional MR approach to explore the causal relationships between gut microbiota and POU as well as MCP. Metabolites are one of the important bridges between gut microbiota and the central nervous system (Eicher and Mohajeri, 2022); thus, MR analysis was also conducted to find the potential associations between metabolites and POU, as well as MCP.

## Materials and methods

### Study design

**Figure 1:**
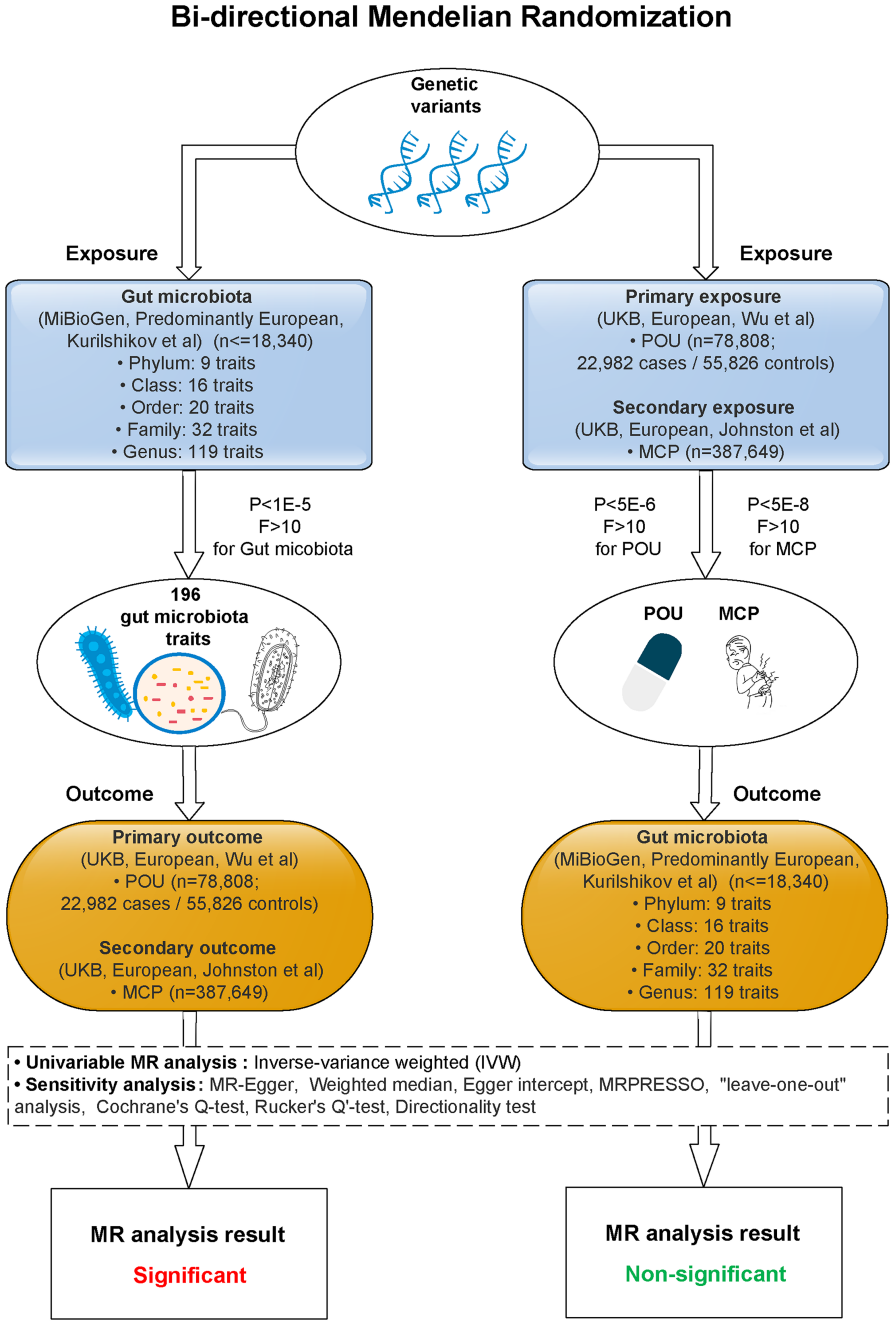
Study flow diagram. This study was a bi-directional Mendelian randomization analysis testing the causal effects between gut microbiota and POU/MCP. All summary-level genetic associations were derived from cohorts of predominant European ancestry. Inverse-variance weighted (IVW) was adopted as the primary method for univariable MR. Sensitivity analyses were adopted to provide robust evidence of MR estimates. Causal effects of 2 gut microbiota traits on POU, and 4 gut microbiota traits on MCP stayed significant after Bonferroni correction (*p* < 0.025, corrected for 2 outcomes). No significant causal association was observed for POU/MCP on gut microbiota after Bonferroni correction (*p* < 2.55 × 10^−4^, corrected for 196 outcomes). MR, Mendelian randomization; POU, prescription opioid use; MCP, multisite chronic pain; F, F statistics; *P*, *p-*value for selected SNPs of exposures.

A two-sample bi-directional designed MR approach was carried out to explore the causality between host genetic-driven gut microbiota and POU, as well as MCP, using summary statistics from large genome-wide association studies (GWASs; Figure 1; Supplementary Table S1). Ethical approval for each GWAS included in this study can be found in the original articles. The study was implemented under Burgess’s guidelines and was reported according to the STROBE-MR statement (Burgess et al., 2019).

### Data sources and instruments

#### Gut microbiota

Summary statistics were leveraged from the largest study of host genetic influences on the abundance of human gut microbiota so far. The MiBioGen consortium curated and analyzed genome-wide genotypes and microbiome data (fecal16S rRNA gene sequencing) from 18,340 individuals (24 cohorts) adjusting for potential covariate effects such as sex, age, BMI, and other covariates. The consortium yielded summary data including 9 phyla, 16 classes, 20 orders, 35 families, and 131 genera, respectively, (Kurilshikov et al., 2021). We discarded those gut microbiota traits that could not be classified, leaving a total of 9 phyla, 16 classes, 20 orders, 32 families, and 119 genera for the MR analysis. Relevant SNPs were identified as having reached the selection threshold of *P* < 1 × 10^−5^, as suggested by Sanna et al. (2019).

#### Prescription opioid use

Summary-level statistics for POU were drawn from a GWAS in United Kingdom Biobank (UKB), comprising a sample size of 78,808 including 22,982 cases and 55,826 controls (Wu et al., 2019). POU was defined as the prescription of active ingredients including but not limited to opioids (e.g., morphine, oxycodone, codeine, fentanyl, pethidine, and tramadol). In this case–control GWAS, Wu et al. identified only three SNPs (single nucleotide polymorphisms) that are robustly related to POU (*p* < 5 × 10^−8^). SNPs with a *P* threshold < 5 × 10^−6^ were used as only a limited number of SNPs meet genome-wide significance.

#### Multisite chronic pain

For chronic pain, we extracted summary statistics from a GWAS in UKB as well. A total of 387,649 participants were enrolled in this study. Johnston et al. performed a GWAS within the United Kingdom general population. Summary data were yielded based on a measure of the number of sites of chronic pain in individuals, adjusting for age, sex, and BMI, and the measure was termed Multisite Chronic Pain (MCP). The definition of MCP is the sum of body sites at which chronic pain (for a duration of at least 3 months). A total of 76 leading SNPs at 39 risk loci were identified to be associated with MCP (Johnston et al., 2019). Only SNPs meeting the genome-wide significance (*p* < 5 × 10^−8^) were selected.

#### Metabolites

Considering that metabolites may play an important role between gut microbiota and host, PubMed was searched for GWASs of metabolites and summary statistics were leveraged from a recent GWAS of the human blood metabolites conducted among European individuals (FHS, *n* = 2,076; Rhee et al., 2013). In this GWAS, Rhee et al. tested all 217 metabolite concentrations present in the datasets adjusting for age, sex, systolic blood pressure, antihypertensive medication use, BMI, diabetes, smoking status, and prevalent cardiovascular disease and renal function. For each metabolite, SNPs were selected at a *P* threshold < 1 × 10^−5^.

### Statistical analysis

Based on the cohort information described in the original GWAS analysis, there was no sample overlapping between exposures and outcomes in this study. Two sample Mendelian randomization analyses were applied as the main statistical method to explore causal relationships between each instrument-exposure and instrument-outcome. To obtain data on independent SNPs, SNPs were clumped and discarded at linkage disequilibrium (LD) r^2^ > 0.001 within a 10,000 kilobase pairs window based on reference data of European ancestry from the 1,000 Genomes Project (Genomes Project, C et al., 2015). SNP effects and relevant standard errors were obtained from GWASs of exposures and outcomes (Hemani et al., 2018b). The F-statistics were calculated to quantify the strength of each SNP. SNPs with F-statistics smaller than 10 were removed. Steiger filtering was carried out on the harmonized data to detect and remove SNPs presenting reverse causation based on the test report. Reverse causation is defined as the observed variance of the outcome outweighing the observed variance of the exposure (Hemani et al., 2018a). The exposure and outcome data were then harmonized, and palindromic SNPs were removed.

After the selection of instrumental variables described above, we performed the MR, using the Inverse-variance weighted (IVW) method as the primary analyses. To provide robust evidence of MR findings, we performed further sensitivity analyses including Weighted median, MR-Egger. The Wald ratio method was applied to get the effect estimate of exposure on outcome for each SNP. The IVW method was then conducted to combine the Wald ratio estimates to obtain a consistent estimate (Pierce and Burgess, 2013). The weighted median assumes that more than half of the instruments are valid (Bowden et al., 2016). On the other hand, the MR-Egger method detects unclear horizontal pleiotropy based on a non-zero intercept value (Bowden et al., 2015). Only if the effect estimates were consistent across the three methods, including IVW, weighted median, and MR-Egger, the results would be enrolled for further sensitivity analyses. To detect any possible pleiotropy, MRPRESSO was also adopted as another method. MRPRESSO is a method to screen horizontal pleiotropy best suited when horizontal pleiotropy occurs in less than 50% of the instruments (Verbanck et al., 2018). Cochran’s *Q*-test, I^2^ statistics, and leave-one-out analysis were applied to detect the heterogeneity of the SNPs that may affect the outcome through an unknown pathway. I^2^ > 25% and *p* < 0.05 in the Cochran *Q*-test were identified as potential existence of heterogeneity (Greco et al., 2015). The Rucker’s Q’-test was also adopted to screen heterogeneity of the MR-Egger model. A minor difference between Q and Q’ (*p* > 0.05) indicated IVW as a better-fitting model (Bowden et al., 2015). An MR Steiger directionality test was carried out to explore whether the causal direction was true (Xue and Pan, 2020). SNPs that exhibit either pleiotropy or heterogeneity were removed according to the results of MRPRESSO and leave-one-out analysis. After that, we repeated MR analyses to obtain the final MR estimates.

For the continuous outcome, the MR effect estimates were reported in beta values (95% CIs), while for the binary outcome, effect estimates were presented in ORs (95% CIs). To adjust multiple comparisons, a Bonferroni correction was applied. *p-*value of effect estimates <0.025 (corrected for 2 outcomes) for the causal effect of gut microbiota on POU and MCP, and 2.55 × 10^−4^ (corrected for 196 outcomes) for the reverse causation, was considered significant. *p-*value smaller than 0.05 but greater than the corrected *p*-value, was considered as a potential causal association. The statistical power of MR estimates was calculated on the website of mRnd.^1^

The MR analyses were performed in the R (version 4.1.2) computing environment using the TwoSampleMR package (version 0.5.6) and the MRPRESSO package (version 1.0). The Effect of the exposure and outcome was harmonized by the TwoSampleMR package, according to comprehensive information on SNPs, like phenotypes, effect alleles, effect allele frequencies, effect sizes, and standard errors for each SNP. In addition, positive-strand alleles and drop palindromic SNPs were inferred according to allele frequencies.

## Results

After clumping and discarding SNPs at linkage disequilibrium (LD) r^2^ > 0.001 within a 10,000 kilobase pairs window, we identified a total of 2,368 and 2,601 SNPs for gut microbiota traits with POU and MCP at the suggestive significance level *p* < 1 × 10^−5^, respectively. Steiger filtering and harmonization were performed after removing SNPs with F-statistics smaller than 10. SNPs that exhibit either pleiotropy or heterogeneity were removed according to the results of MRPRESSO and leave-one-out analysis. At last, 1,999 and 2,188 SNPs were selected for gut microbiota traits with POU and MCP respectively, as shown in Data Sheet 2 in the Supplementary material. After MR analyses, a total of 36 causal associations were identified, including seven gut microbiota traits with POU, five gut microbiota traits with MCP, 12 metabolites traits with POU, and 12 metabolites traits with MCP (Tables 1, 2; Supplementary Tables S4, S5).

**Table 1:** MR results of gut microbiota on prescription opioid use.

							Directional pleiotropy		Cochran Q-test		Rucker’s framework		
Level	Exposure	Outcome	Method	NSNPs	OR (95% CI)	*P*	Egger intercept (*P*)	MRPRESSO global test RSSobs (*P*)	I^2^ statistics	Q-statistic (*P*)	Q’-statistic (*P*)	Q–Q’ (*P*)	Steiger *P*
Genus	Anaerofilum	POU	IVW	9	0.924 (0.857, 0.995)	0.037	0.019 (0.409)	4.527 (0.918)	0.00%	3.617 (0.89)	2.845 (0.899)	0.772 (0.38)	2.55E-40
			Weighted median	9	0.915 (0.83, 1.01)	0.078							
			MR Egger	9	0.785 (0.543, 1.136)	0.241							
Genus	Anaerostipes	POU	IVW	11	0.872 (0.769, 0.989)	0.033	0.021 (0.163)	15.401 (0.366)	7.13%	10.768 (0.376)	8.458 (0.489)	2.311 (0.128)	3.83E-54
			Weighted median	11	0.889 (0.751, 1.052)	0.169							
			MR Egger	11	0.652 (0.439, 0.967)	0.062							
Genus	ChristensenellaceaeR	POU	IVW	8	0.82 (0.709, 0.949)	0.008	0.007 (0.688)	10.228 (0.484)	0.00%	5.838 (0.559)	5.661 (0.462)	0.177 (0.674)	5.17E-28
			Weighted median	8	0.799 (0.664, 0.961)	0.017							
			MR Egger	8	0.755 (0.501, 1.139)	0.230							
Genus	Lachnospiraceae NC2004group	POU	IVW	9	0.917 (0.842, 0.998)	0.046	0.010 (0.643)	10.262 (0.507)	0.00%	6.466 (0.595)	6.231 (0.513)	0.235 (0.628)	5.55E-40
			Weighted median	9	0.917 (0.822, 1.024)	0.123							
			MR Egger	9	0.843 (0.593, 1.198)	0.372							
Genus	Adlercreutzia	POU	IVW	7	1.154 (1.015, 1.313)	0.029	−0.011 (0.687)	15.693 (0.225)	25.65%	8.070 (0.233)	7.785 (0.169)	0.285 (0.594)	1.89E-41
			Weighted median	7	1.073 (0.917, 1.256)	0.378							
			MR Egger	7	1.31 (0.722, 2.375)	0.415							
Genus	Allisonella	POU	IVW	7	1.135 (1.061, 1.213)	0.0002	−0.003 (0.914)	3.940 (0.912)	0.00%	2.863 (0.826)	2.850 (0.723)	0.013 (0.91)	9.79E-36
			Weighted median	7	1.107 (1.01, 1.213)	0.030							
			MR Egger	7	1.163 (0.758, 1.784)	0.520							
Genus	Dialister	POU	IVW	11	1.117 (1.005, 1.242)	0.040	−0.019 (0.266)	13.385 (0.462)	0.00%	8.092 (0.62)	6.685 (0.67)	1.407 (0.236)	2.68E-42
			Weighted median	11	1.064 (0.919, 1.232)	0.408							
			MR Egger	11	1.436 (0.936, 2.205)	0.132							

MR, mendelian randomization; POU, prescription opioid use; IVW, inverse-variance weighted; NSNPs, number of single nucleotide polymorphisms; OR, odds ratio; CI, confidence interval; RSSobs, residual sums of squares of observations.

**Table 2:** MR results of gut microbiota on chronic multisite pain.

							Directional pleiotropy		Cochran Q-test		Rucker’s framework		
Level	Exposure	Outcome	Method	NSNPs	Beta (95% CI)	*P*	Egger intercept (*P*)	MRPRESSO global test RSSobs (*P*)	I^2^ statistics	Q-statistic (*P*)	Q’-statistic (*P*)	Q–Q’ (*P*)	Steiger *P*
Family	Actinomycetaceae	MCP	IVW	5	−0.031 (−0.058, −0.005)	0.020	0.001 (0.804)	4.299 (0.637)	0.00%	2.984 (0.561)	2.910 (0.406)	0.074 (0.786)	2.17E-25
			Weighted median	5	−0.033 (−0.069, 0.003)	0.072							
			MR Egger	5	−0.040 (−0.107, 0.028)	0.331							
Genus	Erysipelotrichaceae UCG003	MCP	IVW	16	−0.029 (−0.050, −0.009)	0.005	0.000 (0.879)	19.059 (0.403)	4.42%	15.694 (0.403)	15.667 (0.334)	0.027 (0.87)	5.61E-81
			Weighted median	16	−0.032 (−0.061, −0.004)	0.025							
			MR Egger	16	−0.034 (−0.092, 0.025)	0.275							
Order	Actinomycetales	MCP	IVW	5	−0.031 (−0.058, −0.005)	0.021	0.001 (0.808)	4.316 (0.645)	0.00%	2.996 (0.559)	2.926 (0.403)	0.070 (0.791)	2.51E-25
			Weighted median	5	−0.033 (−0.066, 0.000)	0.050							
			MR Egger	5	−0.040 (−0.107, 0.028)	0.334							
Family	Clostridiaceae1	MCP	IVW	10	0.031 (0.004, 0.057)	0.023	0.000 (0.926)	13.584 (0.371)	0.00%	7.875 (0.547)	7.866 (0.447)	0.009 (0.923)	9.47E-48
			Weighted median	10	0.026 (−0.012, 0.063)	0.186							
			MR Egger	10	0.027 (−0.048, 0.102)	0.497							
Order	Gastranaerophilales	MCP	IVW	9	0.024 (0.002, 0.045)	0.032	−0.001 (0.811)	18.611 (0.162)	34.39%	12.194 (0.143)	12.088 (0.098)	0.106 (0.745)	1.98E-57
			Weighted median	9	0.018 (−0.007, 0.044)	0.153							
			MR Egger	9	0.031 (−0.033, 0.095)	0.371							

MR, mendelian randomization; MCP, multisite chronic pain; IVW, inverse-variance weighted; NSNPs, number of single nucleotide polymorphisms; Beta, MR effect estimate; CI, confidence interval; RSSobs, residual sums of squares of observations.

### Associations of gut microbiota with POU/MCP

**Figure 2:**
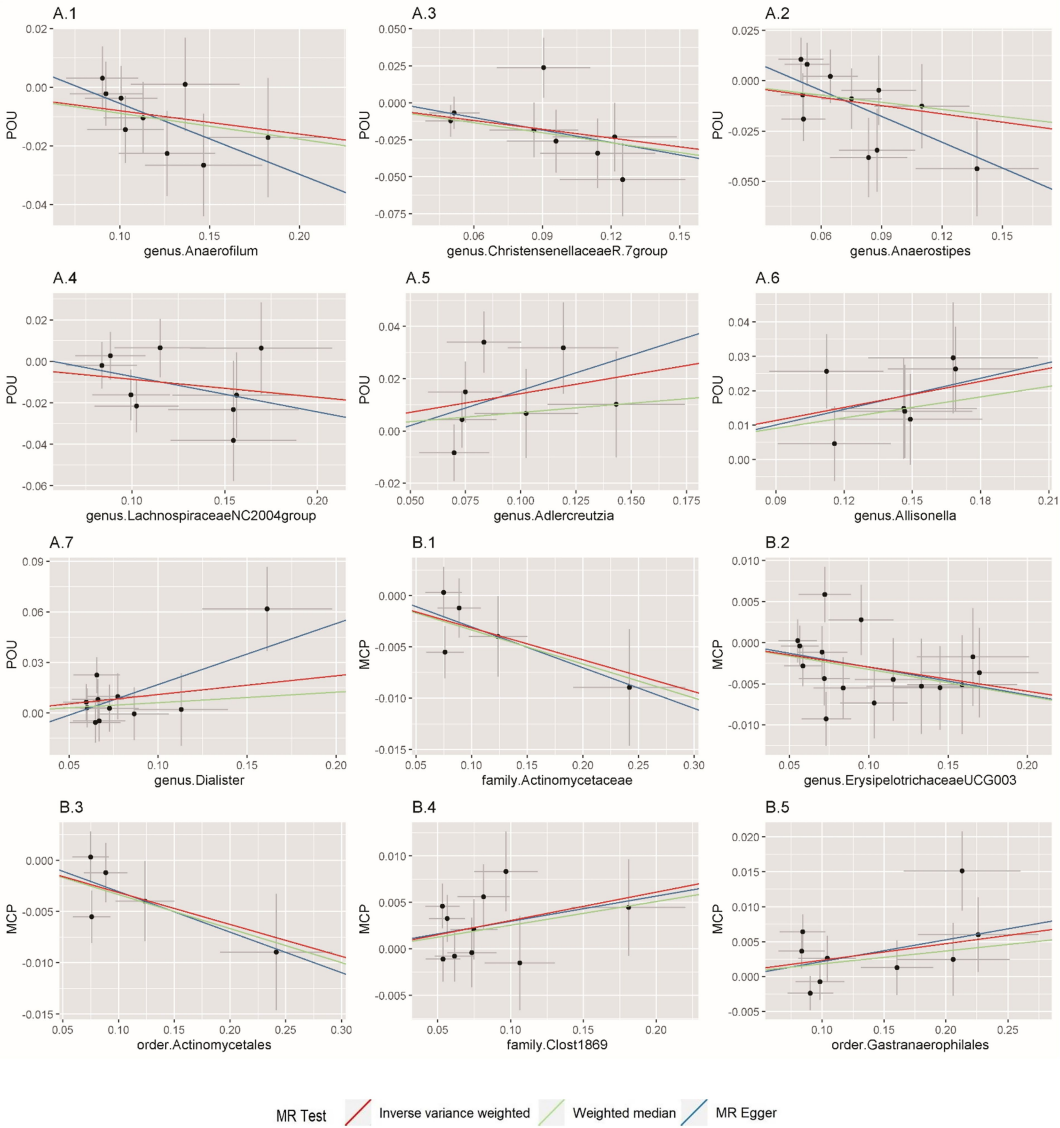
Scatterplot of Mendelian randomization (MR) estimates between prescription opioid use (POU), multisite chronic pain (MCP), and gut microbiota. **(A1–A7)** Associations of gut microbiota traits with POU; **(B1–B5)** Associations of gut microbiota traits with MCP.

In this direction, five positive causal associations were identified, including *Adlercreutzia, Allisonella*, and *Dialister* at the genus level with POU, family *Clostridiaceae1* and order *Gastranaerophilales* with MCP. The host genetic-driven increases in *Adlercreutzia*, *Allisonella*, and *Dialister* at the genus level were potentially related to a higher risk of POU (per relative abundance: *Adlercreutzia* OR = 1.154, 95% CI = 1.015, 1.313, *p* = 0.029; *Allisonella* OR = 1.135, 95% CI = 1.061, 1.213, *p* = 0.0002; *Dialister* OR = 1.117, 95% CI = 1.005, 1.242, *p* = 0.04). Among them, a higher abundance of *Allisonella* at the genus level demonstrated a significantly higher risk of POU causally with a *p*-value of 0.0002 (Table 1). Higher abundances of family *Clostridiaceae1* and order *Gastranaerophilales* were found causally associated with elevated level of MCP (per relative abundance: family *Clostridiaceae1* Beta = 0.031, 95% CI = 0.004, 0.057, *p* = 0.023; order *Gastranaerophilales* Beta = 0.024, 95% CI = 0.002, 0.045, *p* = 0.032; Table 2). As shown in Table 1, a total of four gut microbiota traits showed a negative causal effect on POU, including *LachnospiraceaeNC2004group*, *ChristensenellaceaeR, Anaerostipes*, and *Anaerofilum* at the genus level, whose odds ratios are between 0.820 and 0.924. Order *Actinomycetales*, family *Actinomycetaceae* and genus *ErysipelotrichaceaeUCG003* also showed a protective causal effect on MCP, with regression coefficients between −0.029 and −0.031. Notably, family *Actinomycetaceae* and order *Actinomycetales*, share the same SNPs in the final MR analyses, including rs2889192, rs34583783, rs35011108, rs4073240, and rs58484246, as shown in Data Sheet 2 in the Supplementary material. That is probably because family *Actinomycetaceae* is a sub-category of order *Actinomycetales* and limited loci were identified for these two traits. Scatter plots across various methods are presented in Figure 2.

Considering that chronic intestinal disorders may result in dysbiosis and thus affect the associations between gut microbiota and POU/MCP, several sub-types of chronic bowel disease were identified to check the potential influence. A total of seven traits, including Crohn’s disease, bowel problem, inflammatory bowel disease, irritable bowel syndrome, ulcerative colitis, other non-infective gastroenteritis and colitis, and other functional intestinal disorders were taken into adjustment as suggested by Ni et al. (2021). The associations of the identified IVs with each trait were retrieved in the United Kingdom Biobank summary statistics through the website of GeneATLAS.^2^ As listed in Supplementary Table S2, all *p*-value was above the corrected *p*-value, indicating limited confounding effect of chronic bowel diseases. To further determine whether the identified causal associations were affected by these intestinal diseases, we removed IVs that are relevant to any one of the chronic intestinal disorders at a *P* threshold of <0.05, and reran the MR analysis using the remaining IVs. Similar effect magnitudes were found as compared with the results shown in Tables 1, 2, however, less precisely (Supplementary Table S3). Results of four gut microbiota traits remained significant, including Family *Clostridiaceae1* with MCP (*p* = 0.013), Genus *Allisonella* with POU (*p* = 0.001), Genus *ChristensenellaceaeR.7group* with POU (*p* = 0.008), and Genus *LachnospiraceaeNC2004group* with POU (*p* = 0.021). Meanwhile, sensitivity analyses including the MR-PRESSO test, Cochrane’s Q-test, leave-one-out analysis, MRPRESSO, and MR-Egger intercept showed no evidence of heterogeneity and horizontal pleiotropy (all *p* > 0.05; Supplementary Table S3). Taken together, these results indicated that the above causal associations were unlikely to be mediated by chronic intestinal disorders.

### Associations of POU/MCP with gut microbiota

In this direction, no clear evidence was found for any causal effect of POU on gut microbiota, nor MCP on gut microbiota among all the 196 gut microbiota traits tested.

### Associations of metabolites with POU/MCP

**Figure 3:**
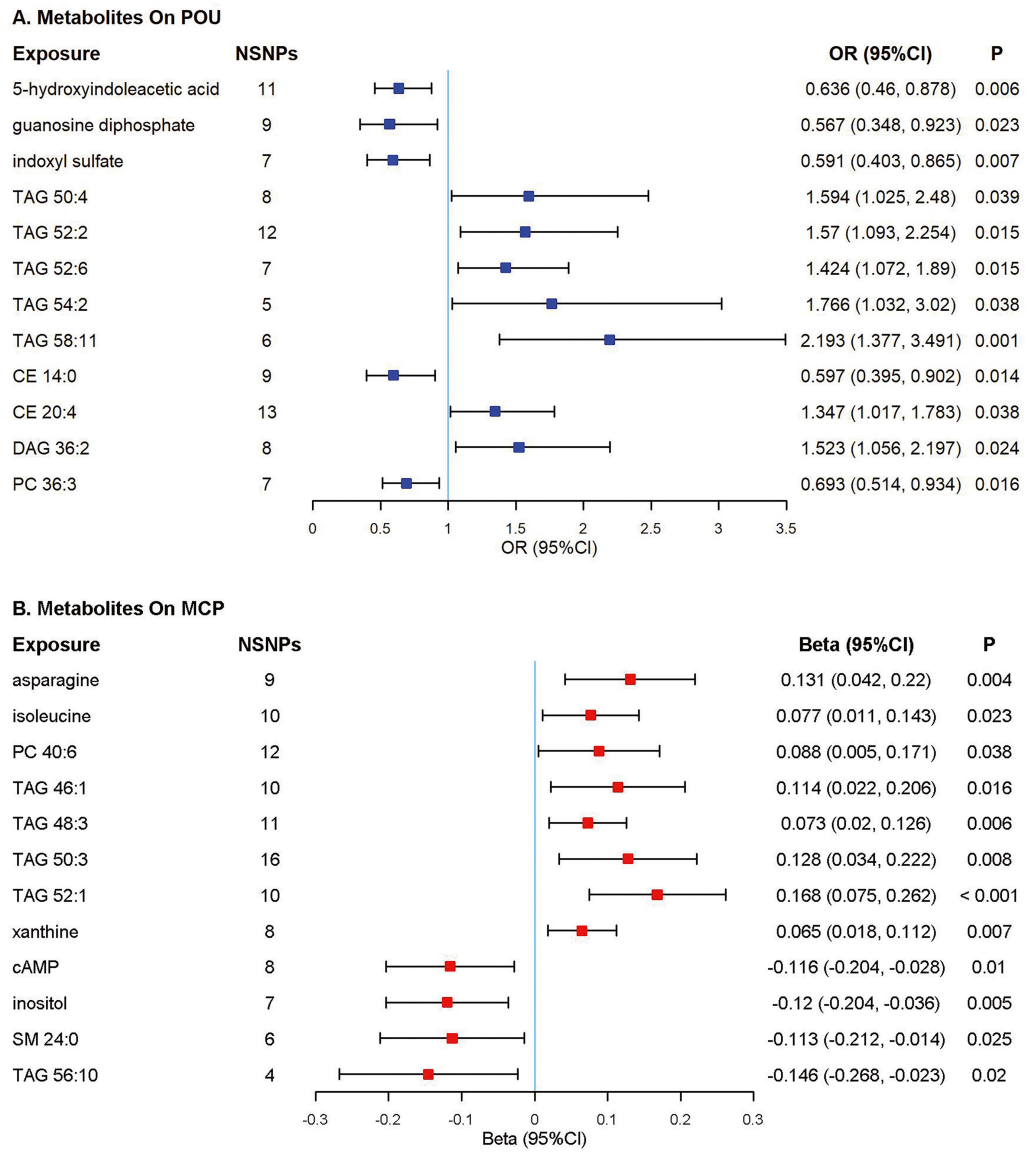
Forest plot of Mendelian randomization (MR) estimates. Causal effects from the inverse-variance weighted Mendelian randomization method. Effect estimates were presented as odds ratios (OR) or beta depending on whether the outcome was binary or continuous, with 95% confidence intervals (CI). **(A)** Odds ratio per 10 units increase in metabolites on prescription opioid use (POU); **(B)** Estimates per 10 units increase in metabolites on multisite chronic pain (MCP). NSNPs, number of single nucleotide polymorphisms; CI, confidence interval; TAG, triacylglycerol; CE, cholesterol ester; DAG, diacylglycerol; PC, phosphatidylcholine; cAMP, cyclic adenosine monophosphate; SM, sphingomyelin.

Among all the 217 metabolites in the MR analyses, host genetic-driven increases in lipid metabolites such as triacylglycerols (TAGs, including TAG 50:4, TAG 52:2, TAG 52:6, TAG 54:2, TAG 58:11), diacylglycerol (DAG 36:2), and cholesterol ester (CE 20:4) were related to a higher risk of POU, with odds ratios ranging from 1.347 to 2.193. Meanwhile, elevated levels of metabolites, including 5-hydroxyindoleacetic acid (5-HIAA), guanosine diphosphate (GDP), indoxyl sulfate, cholesterol ester (CE 14:0) and phosphatidylcholine (PC 36:3) were associated with a lower risk of POU, with odds ratios ranging from 0.567 to 0.693. A total of 9 metabolite traits were found significantly associated with the risk of POU after Bonferroni correction (*p* < 0.025, corrected for 2 outcomes; Figure 3; Supplementary Table S4; Supplementary Figure S2).

Elevated TAG concentrations were found to be potentially associated with higher MCP levels, including TAG 46:1, TAG 48:3, TAG 50:3, and TAG 52:1; while cyclic adenosine monophosphate, inositol and sphingomyelin (SM 24:0) were associated with a lower level of MCP. A total of 10 metabolite traits were found significantly associated with the level of MCP after Bonferroni correction (*p* < 0.025, corrected for 2 outcomes; Figure 3; Supplementary Table S5; Supplementary Figure S3).

### Sensitivity analysis

To provide solid evidence for a true causal association, only if effect estimates were consistent across three MR methods, including IVW, weighted median and MR-Egger, would be enrolled for further sensitivity analysis. No clear evidence of pleiotropy was observed according to the results of MRPRESSO analysis and MR-Egger intercept analysis (all *p* > 0.05). No apparent evidence of overall heterogeneity was identified according to the Cochran’s-Q test (all *p* > 0.05). No single SNP drives the causal association signal according to the leave-one-out sensitivity (Supplementary Figures S1–S3). All the F-statistics for the selected SNPs were larger than 10, indicating an absence of weak instrument bias, as shown in Data Sheet 2 in the Supplementary material. The *p*-value for the MR Steiger directionality test ranged between 10^−16^ and 10^−101^, implying that all the suggestive causal associations were true (Tables 1, 2; Supplementary Tables S3–S5). Power calculation for detecting a significant causal effect (*p* < 0.05) of gut microbiota (OR = 1.2) on the risk of POU, and of gut microbiota on MCP (Beta = 0.1) were almost above 80% (Supplementary Table S6).

All harmonized data were shown in Data Sheet 2 in the Supplementary material. All results of the bi-directional MR analyses between gut microbiota and POU as well as gut microbiota and MCP were shown in Data Sheet 3 in the Supplementary material. All results of the MR analyses between metabolites and POU as well as metabolites and MCP were also shown in Data Sheet 3 in the Supplementary material.

## Discussion

In this study, potential bi-directional associations between host genetic-driven gut microbiota and prescription opioid use (POU), as well as multisite chronic pain (MCP) were evaluated. A total of seven and five gut microbiota traits were identified to be causally associated with POU and MCP, respectively. In the other direction, no clear evidence for any significant causal association was found between POU and gut microbiota as well as MCP and gut microbiota. Besides, metabolites including lipid metabolites and neurotransmitters were found to exhibit causal relationships with POU and MCP.

A total of five positive causal associations were identified, including *Adlercreutzia*, *Allisonella*, and *Dialister* at the genus level with POU, Family *Clostridiaceae1,* and order *Gastranaerophilales* with MCP. Findings from our study were consistent with a cross-sectional study showing that genera *Adlercreutzia* and *Dialister* are significantly related to chronic back pain (Dekker Nitert et al., 2020). The underlying mechanism has not been fully explored; however, it may include the role of gut microbiota in regulating inflammation-driven hyperalgesia, impaired reward behavior, and other neuropsychiatric disorders (Lee et al., 2018; Valles-Colomer et al., 2019; Zhuang et al., 2020). A recent animal study found that gut microbiota and one of its products, valeric acid, may play a crucial role in neuroinflammation(Lai et al., 2021). An increasing number of studies has shown that altered gut microbiota is associated with inflammatory cytokines such as IL-6, IL-8, IFN-γ, and TNF-α (Lin et al., 2019). As is known, inflammatory cytokines play a crucial role in the process of hyperalgesia and thus strengthen the sense of pain, which may also increase the demand for analgesics. Another underlying mechanism is the neuropsychiatric disorders pathway. Many studies found evidence of relationships between gut microbiota and neuropsychiatric diseases and neurodegenerative diseases, for example, major depressive disorder, schizophrenia, and Parkinson’s disease (Zhuang et al., 2020; Rosoff et al., 2021; Ning et al., 2022). One of the crucial symptoms of these diseases is the abnormality in reward processing, which often accompanies with chronic pain and also means increased demand for opioids (Pellissier et al., 2018).

Interestingly, protective causal effects of gut microbiota on POU, as well as gut microbiota on MCP were also found in our study. A total of seven negative relationships were identified, including *Anaerofilum*, *Anaerostipes*, *ChristensenellaceaeR*, and *LachnospiraceaeNC2004group* at the genus level with POU, genus *ErysipelotrichaceaeUCG003*, family *Actinomycetaceae,* and order *Actinomycetales* with MCP. These associations are broadly supported by previous studies. As is known, *Anaerofilum*, *Anaerostipes*, *ChristensenellaceaeR.7group*, and *LachnospiraceaeNC2004group* are all Gram-positive and anaerobic bacterial genera from the class Clostridia and the phylum *Firmicutes*. Class *Clostridia* is a producer of short-chain fatty acids (SCFAs), mainly including butyrate. It is widely recognized that SCFA butyrate may reduce oxidative damages and suppress inflammation, thus alleviating pain perception and further reducing the demand for pain medications (Russo et al., 2018).

In the other direction, no clear evidence was found to reveal any significant causal relationship between POU and gut microbiota nor MCP and gut microbiota. This was contradicted with traditional studies that generally suggest gut microbiota alteration as a result of chronic pain and long-term use of pain medications such as opioids (Banerjee et al., 2016; Acharya et al., 2017), thus supporting the fact that these previous observational associations may be due to confounding or reverse causality. Santanu et al. showed that long-term use of morphine was significantly associated with gut microbiota alterations and resulted in an increase in the abundance of Gram-positive pathogenic bacterial strains (Banerjee et al., 2016). In another cross-sectional study of patients with diabetes, a similar conclusion was yielded that the use of opioids was accompanied by changes in the gut microbiota, including levels of *Bifidobacterium* (Barengolts et al., 2018). In addition, Gicquelais et al. performed a 16S rRNA gene sequencing, using fecal samples from 46 patients in an outpatient addiction clinic. Results from this study showed a decrease in *Roseburia* (butyrate-producing bacteria) and *Bilophila* in the prescription opioid use group but not in the nonopioid control group (Gicquelais et al., 2020). The reason that our results were different from the above-mentioned observational studies may be due to the small sample sizes (ranging from 46 to 99), which were susceptible to unmeasured confounders such as diet and health status, leading to biased conclusions.

Given that metabolites may play a crucial role in the interaction between gut microbiota and POU/MCP, MR analysis was conducted to find their potential associations. It was surprising to find that a series of lipid metabolites may increase the risk of POU. Higher levels of triacylglycerol, diacylglycerol, and cholesterol ester potentially link to the risk of POU and/or MCP, implying their important roles in the microbiota-host crosstalk. These results are consistent with a previous study that showed higher levels of triacylglycerol in patients with chronic pain would increase pain severity (Sibille et al., 2016). Interestingly, prior studies also showed that prebiotics or probiotics could be adopted in the management of lipid metabolites regulation (Barengolts, 2016). Furthermore, a recent MR study also showed a causal relationship between fecal microbiota and triglyceride (TAG) concentration, which strongly indicates that the levels of lipid metabolites, especially TAGs, are associated with gut microbiota composition (Liu et al., 2022). From the MR analysis in this study, a group of neurotransmitters, such as 5-hydroxyindoleacetic acid (5-HIAA), inositol, sphingomyelin, and cyclic adenosine monophosphate, were observed to possibly act as protective factors in reducing the risk of POU or MCP. As the primary metabolite of serotonin, a higher level of 5-HIAA was observed to increase significantly after morphine treatment and had shown to be associated with gut microbiota alteration in animal studies (Guzman et al., 2014; Yue et al., 2021). This may indicate the possible role of 5-HIAA in mediating the interaction between gut microbiota and POU. In this study, an elevated level of inositol was noticed to be potentially associated with a lower risk of MCP. This result coincided with a recent study showing that spliced inositol-requiring enzyme 1[α] (IRE1[α])-X-box-binding protein (XBP1) is a mediator in the process of prostaglandin biosynthesis and plays a role in controlling pain (Chopra et al., 2019). In conclusion, from the perspective of the effects of metabolites on POU and MCP, we believe that lipid metabolites and neurotransmitters may be more worthy of our attention, which may also provide us with another possible way to reduce POU and MCP.

This study has several strengths. Firstly, this is a two-sample bi-directional designed MR analysis to assess the causality between gut microbiota and POU as well as gut microbiota and MCP. This design can minimize the potential bias due to confounding as well as reverse causality in traditional observational studies. Secondly, summary-level data on POU and gut microbiota are the largest GWASs to date and there were no overlapping samples in datasets between exposures and outcomes, which may avoid bias caused by the winner’s curse phenomenon (Bowden and Dudbridge, 2009). Thirdly, since the GWAS study of gut microbiota, POU, and MCP was the largest to date, this study thus has enough statistical power to detect causal effects. The statistical power to identify an odds ratio of 1.2 per 1 relative abundance increment in the gut microbiota on POU with *p* < 0.05 was between 80 and 100%. Fourthly, evidence for causal inference was confirmed by the consistent direction and magnitude of effect estimates across three MR methods, including IVW, weighted median, and MR-Egger. Moreover, the horizontal pleiotropic test including the MR-PRESSO outlier test and MR-Egger intercept did not present any pleiotropy. Cochrane’s Q-test and leave-one-out analysis showed no Heterogeneity. The results of sensitivity analyses provide us with strong evidence of the robustness of MR estimates.

Nevertheless, some limitations should be noted when interpreting findings from this study. To start with, while we leveraged summary statistics from the largest GWAS on POU, gut microbiota, and metabolites, limited SNPs meeting genome-wide significance were identified, which may result in weak genetic instruments. To solve this problem, the *p* value threshold for SNPs was relaxed to (*p* < 5 × 10^−6^) for POU, and (*p* < 1 × 10^−5^) for gut microbiota and metabolites, to include additional SNPs. The F-statistics were then calculated to quantify the strength of each SNP and only SNPs with F-statistics larger than 10 were enrolled for further analysis. Secondly, the biological actions of the selected SNPs were not fully studied, making it hard to identify all potential pleiotropy. However, we ruled out the possible affecting factor of chronic intestine disorders. Furthermore, sensitivity analyses including MRPRESSO and MR Egger intercept did not detect any horizontal pleiotropy in this study. Finally, although the gut microbiota GWAS adopted in this study is the largest at present, nevertheless, its sample size is still relatively small, and the loci studied are relatively limited. So, further studies should be conducted to confirm our results when larger GWAS statistics are available.

## Data availability statement

The datasets presented in this study can be found in online repositories. The names of the repository/repositories and accession number(s) can be found in the article/Supplementary material.

## Author contributions

LL, JL, JZ, and DL were responsible for the concept and design. JQ, FW, XB, and WM assisted with carrying out the analyses. LL and JL drafted the early version of the manuscript. JZ and DL jointly supervised the study. All authors contributed to the article and approved the submitted version.

## Funding

This work was supported by grants from the China Postdoctoral Science Foundation (2021M700176) and the Sun Yat-sen Memorial Hospital Launch Foundation (YXQH2022017).

## Conflict of interest

The authors declare that the research was conducted in the absence of any commercial or financial relationships that could be construed as a potential conflict of interest.

## Publisher’s note

All claims expressed in this article are solely those of the authors and do not necessarily represent those of their affiliated organizations, or those of the publisher, the editors and the reviewers. Any product that may be evaluated in this article, or claim that may be made by its manufacturer, is not guaranteed or endorsed by the publisher.

## Footnotes

^1^ http://cnsgenomics.com/shiny/mRnd/

^2^ http://geneatlas.roslin.ed.ac.uk/phewas

## References

[ref1] Acharya C. Betrapally N. Gillevet P. Sterling R. Akbarali H. White M. . (2017). Chronic opioid use is associated with altered gut microbiota and predicts readmissions in patients with cirrhosis. Aliment. Pharmacol. 45, 319–331. doi: 10.1111/apt.13858, PMID: 27868217

[ref2] Banerjee S. Sindberg G. Wang F. Meng J. Sharma U. Zhang L. . (2016). Opioid-induced gut microbial disruption and bile dysregulation leads to gut barrier compromise and sustained systemic inflammation. Mucosal Immunol. 9, 1418–1428. doi: 10.1038/mi.2016.9, PMID: 26906406 PMC4996771

[ref3] Barengolts E. J. E. P. (2016). Gut microbiota, prebiotics, probiotics, and synbiotics in management of obesity and prediabetes: review of randomized controlled trials. Endocr. Pract. 22, 1224–1234. doi: 10.4158/EP151157.RA, PMID: 27409822

[ref4] Barengolts E. Green S. J. Eisenberg Y. Akbar A. Reddivari B. Layden B. T. . (2018). Gut microbiota varies by opioid use, circulating leptin and oxytocin in African American men with diabetes and high burden of chronic disease. PLoS One 13:e0194171. doi: 10.1371/journal.pone.0194171, PMID: 29596446 PMC5875756

[ref5] Bowden J. Davey Smith G. Burgess S. J. I. J. O. E. (2015). Mendelian randomization with invalid instruments: effect estimation and bias detection through Egger regression. Int. J. Epidemiol. 44, 512–525. doi: 10.1093/ije/dyv080, PMID: 26050253 PMC4469799

[ref6] Bowden J. Davey Smith G. Haycock P. C. Burgess S. J. G. E. (2016). Consistent estimation in Mendelian randomization with some invalid instruments using a weighted median estimator. Genet. Epidemiol. 40, 304–314. doi: 10.1002/gepi.21965, PMID: 27061298 PMC4849733

[ref7] Bowden J. Dudbridge F. J. G. E. T. O. P. O. T. I. G. E. S. (2009). Unbiased estimation of odds ratios: combining genomewide association scans with replication studies. Genet. Epidemiol. 33, 406–418. doi: 10.1002/gepi.20394, PMID: 19140132 PMC2726957

[ref8] Burgess S. Smith G. D. Davies N. M. Dudbridge F. Gill D. Glymour M. M. . (2019). Guidelines for performing Mendelian randomization investigations. Wellcome Open Res. 4:186. doi: 10.12688/wellcomeopenres.15555.1 32760811 PMC7384151

[ref9] Cai J. He L. Wang H. Rong X. Chen M. Shen Q. . (2022). Genetic liability for prescription opioid use and risk of cardiovascular diseases: a multivariable Mendelian randomization study. Addiction 117, 1382–1391. doi: 10.1111/add.15767, PMID: 34859517

[ref10] Chen J. Wang Q. Wang A. Lin Z. (2020). Structural and functional characterization of the gut microbiota in elderly women with migraine. Front. Cell. Infect. Microbiol. 9:470. doi: 10.3389/fcimb.2019.00470, PMID: 32083024 PMC7001586

[ref11] Chopra S. Giovanelli P. Alvarado-Vazquez P. A. Alonso S. Song M. Sandoval T. A. . (2019). IRE1α–XBP1 signaling in leukocytes controls prostaglandin biosynthesis and pain. Science 365:eaau6499. doi: 10.1126/science.aau6499, PMID: 31320508

[ref12] De Vries F. Bruin M. Lobatto D. J. Dekkers O. M. Schoones J. W. Van Furth W. R. . (2020). Opioids and their endocrine effects: a systematic review and meta-analysis. J. Clin. Endocrinol. 105, 1020–1029. doi: 10.1210/clinem/dgz022, PMID: 31511863 PMC7054712

[ref13] Dekker Nitert M. Mousa A. Barrett H. L. Naderpoor N. De Courten B. (2020). Altered gut microbiota composition is associated with back pain in overweight and obese individuals. Front. Endocrinol. 11:605. doi: 10.3389/fendo.2020.00605, PMID: 32982987 PMC7492308

[ref14] Eicher T. P. Mohajeri M. H. (2022). Overlapping mechanisms of action of brain-active bacteria and bacterial metabolites in the pathogenesis of common brain diseases. Nutrients 14:2661. doi: 10.3390/nu14132661, PMID: 35807841 PMC9267981

[ref15] Emdin C. Khera A. Kathiresan S. J. J. (2017). Mendelian Randomization. JAMA 318, 1925–1926. doi: 10.1001/jama.2017.17219 29164242

[ref16] Farmer A. D. Holt C. B. Downes T. J. Ruggeri E. Del Vecchio S. De Giorgio R. J. T. L. G. . (2018). Pathophysiology, diagnosis, and management of opioid-induced constipation. Lancet Gastroenterol. 3, 203–212. doi: 10.1016/S2468-1253(18)30008-6, PMID: 29870734

[ref17] Freidin M. B. Stalteri M. A. Wells P. M. Lachance G. Baleanu A.-F. Bowyer R. C. . (2021). An association between chronic widespread pain and the gut microbiome. Rheumatology 60, 3727–3737. doi: 10.1093/rheumatology/keaa847, PMID: 33331911 PMC8328510

[ref18] Genomes Project, C Auton A. Brooks L. D. Durbin R. M. Garrison E. P. Kang H. M. . (2015). A global reference for human genetic variation. Nature 526, 68–74. doi: 10.1038/nature15393 26432245 PMC4750478

[ref19] Gicquelais R. E. Bohnert A. S. Thomas L. Foxman B. (2020). Opioid agonist and antagonist use and the gut microbiota: associations among people in addiction treatment. Sci. Rep. 10, 1–11. doi: 10.1038/s41598-020-76570-9 33173098 PMC7655955

[ref20] Greco M. F. Minelli C. Sheehan N. A. Thompson J. R. (2015). Detecting pleiotropy in Mendelian randomisation studies with summary data and a continuous outcome. Stat. Med. 34, 2926–2940. doi: 10.1002/sim.6522, PMID: 25950993

[ref21] Guida F. Boccella S. Belardo C. Iannotta M. Piscitelli F. De Filippis F. . (2020). Altered gut microbiota and endocannabinoid system tone in vitamin D deficiency-mediated chronic pain. Brain Behav. Immun. 85, 128–141. doi: 10.1016/j.bbi.2019.04.006, PMID: 30953765

[ref22] Guzman D. C. Garcia E. H. Mejia G. B. Olguin H. J. Gonzalez J. Na L. R. J. P. J. O. B. S. P. (2014). Effect of morphine and lacosamide on levels of dopamine and 5-HIAA in brain regions of rats with induced hypoglycemia. Pak. J. Biol. Sci. 17, 292–296. doi: 10.3923/pjbs.2014.292.296, PMID: 24783817

[ref23] Hadland S. E. Bagley S. M. Gai M. J. Earlywine J. J. Schoenberger S. F. Morgan J. R. . (2021). Opioid use disorder and overdose among youth following an initial opioid prescription. Addiction 116, 2790–2800. doi: 10.1111/add.15487, PMID: 33739476 PMC8429061

[ref24] Hemani G. Bowden J. Davey Smith G. J. H. M. G. (2018a). Evaluating the potential role of pleiotropy in Mendelian randomization studies. Hum. Mol. Genet. 27, R195–R208. doi: 10.1093/hmg/ddy163, PMID: 29771313 PMC6061876

[ref25] Hemani G. Zheng J. Elsworth B. Wade K. Haberland V. Baird D. . (2018b). The MR-base platform supports systematic causal inference across the human phenome. Elife 7:e34408. doi: 10.7554/eLife.34408, PMID: 29846171 PMC5976434

[ref26] Johnston K. J. Adams M. J. Nicholl B. I. Ward J. Strawbridge R. J. Ferguson A. . (2019). Genome-wide association study of multisite chronic pain in UK biobank. PLoS Genet. 15:e1008164. doi: 10.1371/journal.pgen.1008164, PMID: 31194737 PMC6592570

[ref27] Kang M. Mischel R. A. Bhave S. Komla E. Cho A. Huang C. . (2017). The effect of gut microbiome on tolerance to morphine mediated antinociception in mice. Sci. Rep. 7, 1–17. doi: 10.1038/srep42658 28211545 PMC5314392

[ref28] Kurilshikov A. Medina-Gomez C. Bacigalupe R. Radjabzadeh D. Wang J. Demirkan A. . (2021). Large-scale association analyses identify host factors influencing human gut microbiome composition. Nat. Genet. 53, 156–165. doi: 10.1038/s41588-020-00763-1, PMID: 33462485 PMC8515199

[ref29] Lai Z. Shan W. Li J. Min J. Zeng X. Zuo Z. (2021). Appropriate exercise level attenuates gut dysbiosis and valeric acid increase to improve neuroplasticity and cognitive function after surgery in mice. Mol. Psychiatry 26, 7167–7187. doi: 10.1038/s41380-021-01291-y, PMID: 34663905 PMC8873004

[ref30] Lee K. Vuong H. Nusbaum D. Hsiao E. Evans C. Taylor A. J. N. O. P. O. T. A. C. O. N. (2018). The gut microbiota mediates reward and sensory responses associated with regimen-selective morphine dependence. Neuropsychopharmacology 43, 2606–2614. doi: 10.1038/s41386-018-0211-9, PMID: 30258112 PMC6224506

[ref31] Lin C.-H. Chen C.-C. Chiang H.-L. Liou J.-M. Chang C.-M. Lu T.-P. . (2019). Altered gut microbiota and inflammatory cytokine responses in patients with Parkinson’s disease. J. Neuroinflammation 16, 1–9. doi: 10.1186/s12974-019-1528-y 31248424 PMC6598278

[ref32] Liu X. Tong X. Zou Y. Lin X. Zhao H. Tian L. . (2022). Mendelian randomization analyses support causal relationships between blood metabolites and the gut microbiome. Nat. Genet. 54, 52–61. doi: 10.1038/s41588-021-00968-y 34980918

[ref33] Meng J. Banerjee S. Li D. Sindberg G. M. Wang F. Ma J. . (2015). Opioid exacerbation of gram-positive sepsis, induced by gut microbial modulation, is rescued by IL-17A neutralization. Sci. Rep. 5, 1–17. doi: 10.1038/srep10918 PMC4454150 26039416

[ref34] Meng J. Yu H. Ma J. Wang J. Banerjee S. Charboneau R. . (2013). Morphine induces bacterial translocation in mice by compromising intestinal barrier function in a TLR-dependent manner. PLoS One 8:e54040. doi: 10.1371/journal.pone.0054040, PMID: 23349783 PMC3548814

[ref35] Newlove-Delgado T. Abbott R. A. Martin A. E. J. J. P. (2019). Probiotics for children with recurrent abdominal pain. JAMA Pediatr. 173, 183–184. doi: 10.1001/jamapediatrics.2018.4575, PMID: 30592480

[ref36] Ni J.-J. Xu Q. Yan S.-S. Han B.-X. Zhang H. Wei X.-T. . (2021). Gut microbiota and psychiatric disorders: a two-sample Mendelian randomization study. Front. Microbiol. 12:737197. doi: 10.3389/fmicb.2021.737197 35185808 PMC8856606

[ref37] Ning J. Huang S.-Y. Chen S.-D. Zhang Y.-R. Huang Y.-Y. Yu J.-T. (2022). Investigating casual associations among gut microbiota, metabolites, and neurodegenerative diseases: a Mendelian randomization study. J. Alzheimers Dis. 87, 211–222. doi: 10.3233/JAD-215411 35275534

[ref38] Ochoa-Repáraz J. Ramelow C. C. Kasper L. H. (2020). A gut feeling: the importance of the intestinal microbiota in psychiatric disorders. Front. Immunol. 2735:510113. doi: 10.3389/fimmu.2020.510113 PMC7604426 33193297

[ref39] Pellissier L. P. Gandía J. Laboute T. Becker J. A. Le Merrer J. (2018). μ opioid receptor, social behaviour and autism spectrum disorder: reward matters. Br. J. Pharmacol. 175, 2750–2769. doi: 10.1111/bph.13808, PMID: 28369738 PMC6016638

[ref40] Pierce B. L. Burgess S. J. A. J. O. E. (2013). Efficient design for Mendelian randomization studies: subsample and 2-sample instrumental variable estimators. Am. J. Epidemiol. 178, 1177–1184. doi: 10.1093/aje/kwt084, PMID: 23863760 PMC3783091

[ref41] Rhee E. P. Ho J. E. Chen M.-H. Shen D. Cheng S. Larson M. G. . (2013). A genome-wide association study of the human metabolome in a community-based cohort. Cell Metab. 18, 130–143. doi: 10.1016/j.cmet.2013.06.013, PMID: 23823483 PMC3973158

[ref42] Rosoff D. B. Smith G. D. Lohoff F. W. J. J. P. (2021). Prescription opioid use and risk for major depressive disorder and anxiety and stress-related disorders: a multivariable mendelian randomization analysis. JAMA Psychiat. 78, 151–160. doi: 10.1001/jamapsychiatry.2020.3554, PMID: 33175090 PMC7658804

[ref43] Russo R. Cristiano C. Avagliano C. De Caro C. La Rana G. Raso G. M. . (2018). Gut-brain axis: role of lipids in the regulation of inflammation, pain and CNS diseases. Curr. Med. Chem. 25, 3930–3952. doi: 10.2174/0929867324666170216113756, PMID: 28215162

[ref44] Sandhu H. Underwood M. Furlan A. Noyes J. Eldabe S. (2018). What interventions are effective to taper opioids in patients with chronic pain? BMJ 362:k2990. doi: 10.1136/bmj.k2990 30262590

[ref45] Sanna S. Van Zuydam N. R. Mahajan A. Kurilshikov A. Vich Vila A. Võsa U. . (2019). Causal relationships among the gut microbiome, short-chain fatty acids and metabolic diseases. Nat. Genet. 51, 600–605. doi: 10.1038/s41588-019-0350-x, PMID: 30778224 PMC6441384

[ref46] Sibille K. T. Steingrímsdóttir Ó. A. Fillingim R. B. Stubhaug A. Schirmer H. Chen H. . (2016). Investigating the burden of chronic pain: an inflammatory and metabolic composite. Pain Res. 2016:7657329. doi: 10.1155/2016/7657329 PMC4909918 27445627

[ref48] Valles-Colomer M. Falony G. Darzi Y. Tigchelaar E. F. Wang J. Tito R. Y. . (2019). The neuroactive potential of the human gut microbiota in quality of life and depression. Nat. Microbiol. 4, 623–632. doi: 10.1038/s41564-018-0337-x, PMID: 30718848

[ref49] Verbanck M. Chen C.-Y. Neale B. Do R. J. N. G. (2018). Detection of widespread horizontal pleiotropy in causal relationships inferred from Mendelian randomization between complex traits and diseases. Nat. Genet. 50, 693–698. doi: 10.1038/s41588-018-0099-7, PMID: 29686387 PMC6083837

[ref50] Wang F. Meng J. Zhang L. Johnson T. Chen C. Roy S. J. S. R. (2018). Morphine induces changes in the gut microbiome and metabolome in a morphine dependence model. Sci. Rep. 8, 1–15. doi: 10.1038/s41598-018-21915-8 29483538 PMC5827657

[ref51] Wu Y. Byrne E. M. Zheng Z. Kemper K. E. Yengo L. Mallett A. J. . (2019). Genome-wide association study of medication-use and associated disease in the UK biobank. Nat. Commun. 10, 1–10. doi: 10.1038/s41467-019-09572-5 31015401 PMC6478889

[ref52] Xue H. Pan W. J. P. G. (2020). Inferring causal direction between two traits in the presence of horizontal pleiotropy with GWAS summary data. PLoS Genet. 16:e1009105. doi: 10.1371/journal.pgen.1009105, PMID: 33137120 PMC7660933

[ref53] Yang C. Fang X. Zhan G. Huang N. Li S. Bi J. . (2019). Key role of gut microbiota in anhedonia-like phenotype in rodents with neuropathic pain. Transl. Psychiatry 9, 1–11. doi: 10.1038/s41398-019-0379-8 30705252 PMC6355832

[ref54] Yue Q. Cai M. Xiao B. Zhan Q. Zeng C. J. F. I. N. (2021). A high-tryptophan diet reduces seizure-induced respiratory arrest and alters the gut microbiota in DBA/1 mice. Front. Neurol. 12:762323. doi: 10.3389/fneur.2021.762323, PMID: 34887831 PMC8650499

[ref55] Zhang L. Meng J. Ban Y. Jalodia R. Chupikova I. Fernandez I. . (2019). Morphine tolerance is attenuated in germfree mice and reversed by probiotics, implicating the role of gut microbiome. Proc. Natl. Acad. Sci. 116, 13523–13532. doi: 10.1073/pnas.1901182116, PMID: 31209039 PMC6613141

[ref56] Zhang L. Roy S. J. C. S. H. P. I. M. (2021). Opioid modulation of the gut–brain Axis in opioid-associated comorbidities. Cold Spring Harb. Perspect. Med. 11:a040485. doi: 10.1101/cshperspect.a040485, PMID: 32816876 PMC8415294

[ref57] Zhuang Z. Yang R. Wang W. Qi L. Huang T. J. J. O. N. (2020). Associations between gut microbiota and Alzheimer’s disease, major depressive disorder, and schizophrenia. J. Neuroinflammation 17, 1–9. doi: 10.1186/s12974-020-01961-8 33008395 PMC7532639

