# Supplementary material for: Gut microbiota alterations may increase the risk of prescription opioid use, but not vice versa: A two-sample bi-directional Mendelian randomization study
Source: Front Microbiol. 2022 Nov 22;13:994170. doi: 10.3389/fmicb.2022.994170 (PMC9722965; doi:10.3389/fmicb.2022.994170)
Supplement: Supplementary file 1 [file Data_Sheet_1.DOCX]

**Supplementary Materials for**

**This file includes:**

**Table S1.** Description of prescription opioid use, multisite chronic pain, gut microbiota, and metabolites.

**Table S2.** POU/MCP associated SNPs and traits of chronic bowel or intestine disease.

**Table S3.** MR Results of gut microbiota on POU/MCP after removing SNPs associated with traits of chronic bowel or intestine disease.

**Table S4.** MR Results of metabolites on POU.

**Table S5.** MR Results of metabolites on MCP.

**Table S6.** MR Power calculation for detecting significant (P < 0.05) causal effect (OR = 1.2) of gut microbiota on the risk of POU, and (beta = 0.1) of gut microbiota on MCP.

**Figure S1.** Leave-one-out analysis of inverse-variance weighted (IVW) estimates between genetically determined gut microbiota traits (p < 1 × 10^-5^) and prescription opioid use (POU), as well as multisite chronic pain (MCP).

**Figure S2.** Leave-one-out analysis of inverse-variance weighted (IVW) estimates between genetically determined metabolites traits (p < 1 × 10^-5^) and prescription opioid use (POU).

**Figure S3.** Leave-one-out analysis of inverse-variance weighted (IVW) estimates between genetically determined metabolites traits (p < 1 × 10^-5^) and multisite chronic pain (MCP).

**Additional information 1.** Harmonized data**.**

**Additional information 2.** Results of MR analysis.

**Table S1. Description of prescription opioid use, multisite chronic pain, gut microbiota and metabolite.**

| **Traits** | **Consortium** | **First author** | **Sample size** | **Populations** | **Journal** | **Year** | **Download link** |
| --- | --- | --- | --- | --- | --- | --- | --- |
| Prescription opioid use | NA | Wu | 78,808  (22,982 cases / 55,826 controls) | European ancestry | [Nat Commun.](https://www.ncbi.nlm.nih.gov/pmc/articles/PMC6478889/) | 2019 | http://ftp.ebi.ac.uk/pub/databases/gwas/summary_statistics/GCST007001-GCST008000/GCST007936/ |
| Multisite chronic pain | NA | Johnston | 387,649 | European ancestry | PLoS Genet. | 2019 | http://ftp.ebi.ac.uk/pub/databases/gwas/summary_statistics/GCST008001-GCST009000/GCST008512/ |
| Gut microbiota | MiBioGen^a^ | Kurilshikov | 18,340 | predominantly European ancestry | Nat Genet. | 2020 | https://mibiogen.gcc.rug.nl/ |
| Metabolites | FHS | Rhee | 2076 | European ancestry | Cell Metab. | 2013 | https://www.cell.com/cell-metabolism/fulltext/S1550-4131(13)00257-X?_returnURL=https%3A%2F%2Flinkinghub.elsevier.com%2Fretrieve%2Fpii%2FS155041311300257X%3Fshowall%3Dtrue#secsectitle0110 |
| ^a^ MiBioGen consortium curated and analyzed genome-wide genotypes and 16S fecal microbiome data from 18,340 individuals (24 cohorts). Twenty cohorts included samples of single ancestry, namely European (16 cohorts; n= 13,266), Middle Eastern (1 cohort; n= 481), East Asian (1 cohort; n= 811), American Hispanic/Latin (1 cohort; n= 1,097) and African American (1 cohort; n= 114), whereas four cohorts included samples from multiple ancestries (n = 2,571). | | | | | | | |

**Table S2. POU/MCP associated SNPs and traits of chronic bowel or intestine disease.**

| **Bacterial taxa (exposure)** | **Outcome** | **SNP** | **Irritable bowel syndrome** | **Inflammatory bowel disease** | **Ulcerative colitis** | **Crohn's disease** | **Bowel problem** | **Other non-infective gastroenteritis and colitis** | **Other functional intestinal disorders** |
| --- | --- | --- | --- | --- | --- | --- | --- | --- | --- |
| genus.Adlercreutzia.id.812 | POU | rs11604400 | 0.135 | 0.415 | 0.240 | 0.003 | 0.569 | 0.249 | 0.508 |
| genus.Adlercreutzia.id.812 | POU | rs13231526 | 0.244 | 0.438 | 0.092 | 0.278 | 0.170 | 0.707 | 0.836 |
| genus.Adlercreutzia.id.812 | POU | rs2717140 | 0.027 | 0.065 | 0.039 | 0.731 | 0.170 | 0.835 | 0.830 |
| genus.Adlercreutzia.id.812 | POU | rs55719207 | 0.136 | 0.445 | 0.928 | 0.664 | 0.113 | 0.568 | 0.997 |
| genus.Adlercreutzia.id.812 | POU | rs7680684 | 0.134 | 0.458 | 0.145 | 0.593 | 0.999 | 0.159 | 0.418 |
| genus.Adlercreutzia.id.812 | POU | rs9490822 | 0.786 | 0.666 | 0.114 | 0.395 | 0.567 | 0.958 | 0.952 |
| genus.Adlercreutzia.id.812 | POU | rs9915817 | 0.320 | 0.960 | 0.496 | 0.408 | 0.330 | 0.079 | 0.815 |
| genus.Allisonella.id.2174 | POU | rs1901739 | 0.645 | 0.239 | 0.884 | 0.925 | 0.039 | 0.771 | 0.608 |
| genus.Allisonella.id.2174 | POU | rs35110698 | 0.574 | 0.294 | 0.616 | 0.961 | 0.679 | 0.895 | 0.052 |
| genus.Allisonella.id.2174 | POU | rs35778461 | 0.389 | 0.163 | 0.621 | 0.426 | 0.337 | 0.542 | 0.436 |
| genus.Allisonella.id.2174 | POU | rs594561 | 0.789 | 0.797 | 0.019 | 0.179 | 0.666 | 0.660 | 0.266 |
| genus.Allisonella.id.2174 | POU | rs602075 | 0.329 | 0.078 | 0.058 | 0.237 | 0.303 | 0.979 | 0.267 |
| genus.Allisonella.id.2174 | POU | rs6742198 | 0.598 | 0.818 | 0.311 | 0.094 | 0.619 | 0.986 | 0.800 |
| genus.Allisonella.id.2174 | POU | rs7898615 | 0.112 | 0.959 | 0.589 | 0.776 | 0.706 | 0.404 | 0.918 |
| genus.Dialister.id.2183 | POU | rs10138457 | 0.708 | 0.322 | 0.605 | 0.934 | 0.979 | 0.390 | 0.436 |
| genus.Dialister.id.2183 | POU | rs10938938 | 0.068 | 0.162 | 0.983 | 0.864 | 0.801 | 0.987 | 0.062 |
| genus.Dialister.id.2183 | POU | rs11071887 | 0.944 | 0.159 | 0.452 | 0.103 | 0.449 | 0.545 | 0.169 |
| genus.Dialister.id.2183 | POU | rs11166701 | 0.512 | 0.999 | 0.850 | 0.546 | 0.116 | 0.386 | 0.199 |
| genus.Dialister.id.2183 | POU | rs2314294 | 0.267 | 0.040 | 0.412 | 0.359 | 0.140 | 0.357 | 0.229 |
| genus.Dialister.id.2183 | POU | rs2435610 | 0.753 | 0.168 | 0.603 | 0.256 | 0.946 | 0.579 | 0.764 |
| genus.Dialister.id.2183 | POU | rs4747450 | 0.267 | 0.129 | 0.918 | 0.481 | 0.022 | 0.462 | 0.872 |
| genus.Dialister.id.2183 | POU | rs4753063 | 0.123 | 0.490 | 0.462 | 0.962 | 0.329 | 0.769 | 0.995 |
| genus.Dialister.id.2183 | POU | rs75416973 | 0.712 | 0.254 | 0.276 | 0.606 | 0.416 | 0.558 | 0.694 |
| genus.Dialister.id.2183 | POU | rs764177 | 0.606 | 0.255 | 0.208 | 0.598 | 0.955 | 0.230 | 0.989 |
| genus.Dialister.id.2183 | POU | rs76680460 | 0.002 | 0.527 | 0.957 | 0.299 | 0.690 | 0.694 | 0.947 |
| genus.Anaerofilum.id.2053 | POU | rs1563175 | 0.839 | 0.001 | 0.067 | 0.494 | 0.690 | 0.346 | 0.529 |
| genus.Anaerofilum.id.2053 | POU | rs17012738 | 0.908 | 0.895 | 0.808 | 0.508 | 0.264 | 0.695 | 0.806 |
| genus.Anaerofilum.id.2053 | POU | rs17096874 | 0.936 | 0.887 | 0.487 | 0.568 | 0.627 | 0.113 | 0.839 |
| genus.Anaerofilum.id.2053 | POU | rs4244069 | 0.817 | 0.454 | 0.339 | 0.504 | 0.930 | 0.066 | 0.815 |
| genus.Anaerofilum.id.2053 | POU | rs4506496 | 0.274 | 0.103 | 0.013 | 0.213 | 0.847 | 0.831 | 0.354 |
| genus.Anaerofilum.id.2053 | POU | rs712981 | 0.244 | 0.258 | 0.316 | 0.762 | 0.901 | 0.937 | 0.376 |
| genus.Anaerofilum.id.2053 | POU | rs79598899 | 0.936 | 0.807 | 0.425 | 0.022 | 0.505 | 0.788 | 0.466 |
| genus.Anaerofilum.id.2053 | POU | rs816292 | 0.118 | 0.462 | 0.522 | 0.478 | 0.248 | 0.417 | 0.078 |
| genus.Anaerofilum.id.2053 | POU | rs9299345 | 0.187 | 0.561 | 0.451 | 0.072 | 0.369 | 0.097 | 0.775 |
| genus.Anaerostipes.id.1991 | POU | rs10502061 | 0.910 | 0.245 | 0.379 | 0.872 | 0.620 | 0.738 | 0.511 |
| genus.Anaerostipes.id.1991 | POU | rs2396460 | 0.319 | 0.038 | 0.888 | 0.294 | 0.065 | 0.486 | 0.912 |
| genus.Anaerostipes.id.1991 | POU | rs2804244 | 0.675 | 0.137 | 0.084 | 0.248 | 0.997 | 0.166 | 0.432 |
| genus.Anaerostipes.id.1991 | POU | rs3900776 | 0.361 | 0.514 | 0.168 | 0.977 | 0.763 | 0.536 | 0.540 |
| genus.Anaerostipes.id.1991 | POU | rs62157625 | 0.610 | 0.535 | 0.964 | 0.705 | 0.553 | 0.908 | 0.015 |
| genus.Anaerostipes.id.1991 | POU | rs62215703 | 0.756 | 0.036 | 0.689 | 0.165 | 0.902 | 0.327 | 0.242 |
| genus.Anaerostipes.id.1991 | POU | rs6474958 | 0.177 | 0.636 | 0.771 | 0.421 | 0.321 | 0.636 | 0.494 |
| genus.Anaerostipes.id.1991 | POU | rs6726833 | 0.019 | 0.341 | 0.033 | 0.914 | 0.150 | 0.753 | 0.117 |
| genus.Anaerostipes.id.1991 | POU | rs6854026 | 0.526 | 0.416 | 0.028 | 0.966 | 0.838 | 0.752 | 0.713 |
| genus.Anaerostipes.id.1991 | POU | rs7193624 | 0.141 | 0.525 | 0.025 | 0.235 | 0.026 | 0.854 | 0.221 |
| genus.Anaerostipes.id.1991 | POU | rs78735375 | 0.720 | 0.791 | 0.795 | 0.536 | 0.677 | 0.337 | 0.519 |
| genus.ChristensenellaceaeR.7group.id.11283 | POU | rs17081797 | 0.840 | 0.335 | 0.741 | 0.289 | 0.158 | 0.018 | 0.263 |
| genus.ChristensenellaceaeR.7group.id.11283 | POU | rs60954665 | 0.949 | 0.061 | 0.096 | 0.095 | 0.664 | 0.365 | 0.794 |
| genus.ChristensenellaceaeR.7group.id.11283 | POU | rs62190261 | 0.632 | 0.201 | 0.067 | 0.664 | 0.026 | 0.346 | 0.787 |
| genus.ChristensenellaceaeR.7group.id.11283 | POU | rs62467127 | 0.813 | 0.039 | 0.086 | 0.258 | 0.050 | 0.904 | 0.143 |
| genus.ChristensenellaceaeR.7group.id.11283 | POU | rs73952017 | 0.201 | 0.849 | 0.306 | 0.592 | 0.791 | 0.423 | 0.741 |
| genus.ChristensenellaceaeR.7group.id.11283 | POU | rs78521377 | 0.370 | 0.152 | 0.586 | 0.464 | 0.196 | 0.669 | 0.065 |
| genus.ChristensenellaceaeR.7group.id.11283 | POU | rs79150079 | 0.456 | 0.492 | 0.473 | 0.885 | 0.259 | 0.370 | 0.558 |
| genus.ChristensenellaceaeR.7group.id.11283 | POU | rs892686 | 0.789 | 0.701 | 0.467 | 0.374 | 0.192 | 0.212 | 0.346 |
| genus.LachnospiraceaeNC2004group.id.11316 | POU | rs117467633 | 0.521 | 0.541 | 0.047 | 0.205 | 0.087 | 0.231 | 0.790 |
| genus.LachnospiraceaeNC2004group.id.11316 | POU | rs12127733 | 0.491 | 0.314 | 0.122 | 0.384 | 0.478 | 0.639 | 0.580 |
| genus.LachnospiraceaeNC2004group.id.11316 | POU | rs12208226 | 0.494 | 0.545 | 0.163 | 0.152 | 0.329 | 0.962 | 0.708 |
| genus.LachnospiraceaeNC2004group.id.11316 | POU | rs12863463 | 0.361 | 0.537 | 0.101 | 0.425 | 0.935 | 0.519 | 0.366 |
| genus.LachnospiraceaeNC2004group.id.11316 | POU | rs17067076 | 0.198 | 0.476 | 0.554 | 0.466 | 0.948 | 0.956 | 0.838 |
| genus.LachnospiraceaeNC2004group.id.11316 | POU | rs1928659 | 0.477 | 0.952 | 0.051 | 0.087 | 0.874 | 0.211 | 0.470 |
| genus.LachnospiraceaeNC2004group.id.11316 | POU | rs1929743 | 0.971 | 0.041 | 0.415 | 0.405 | 0.759 | 0.852 | 0.059 |
| genus.LachnospiraceaeNC2004group.id.11316 | POU | rs3756315 | 0.288 | 0.783 | 0.185 | 0.772 | 0.279 | 0.537 | 0.337 |
| genus.LachnospiraceaeNC2004group.id.11316 | POU | rs6116753 | 0.734 | 0.893 | 0.553 | 0.775 | 0.587 | 0.262 | 0.853 |
| family.Actinomycetaceae.id.421 | MCP | rs2889192 | 0.734 | 0.172 | 0.350 | 0.192 | 0.276 | 0.290 | 0.924 |
| family.Actinomycetaceae.id.421 | MCP | rs34583783 | 0.376 | 0.659 | 0.784 | 0.051 | 0.681 | 0.216 | 0.764 |
| family.Actinomycetaceae.id.421 | MCP | rs35011108 | 0.704 | 0.896 | 0.692 | 0.942 | 0.290 | 0.730 | 0.644 |
| family.Actinomycetaceae.id.421 | MCP | rs4073240 | 0.165 | 0.731 | 0.918 | 0.592 | 0.449 | 0.222 | 0.568 |
| family.Actinomycetaceae.id.421 | MCP | rs58484246 | 0.989 | 0.023 | 0.001 | 0.316 | 0.736 | 0.013 | 0.017 |
| genus.ErysipelotrichaceaeUCG003.id.11384 | MCP | rs10164067 | 0.325 | 0.385 | 0.214 | 0.018 | 0.056 | 0.664 | 0.053 |
| genus.ErysipelotrichaceaeUCG003.id.11384 | MCP | rs11666127 | 0.351 | 0.481 | 0.894 | 0.230 | 0.466 | 0.862 | 0.014 |
| genus.ErysipelotrichaceaeUCG003.id.11384 | MCP | rs11994308 | 0.280 | 0.468 | 0.530 | 0.465 | 0.242 | 0.094 | 0.537 |
| genus.ErysipelotrichaceaeUCG003.id.11384 | MCP | rs12251396 | 0.311 | 0.048 | 0.023 | 0.327 | 0.502 | 0.535 | 0.601 |
| genus.ErysipelotrichaceaeUCG003.id.11384 | MCP | rs17798136 | NA | NA | NA | NA | NA | NA | NA |
| genus.ErysipelotrichaceaeUCG003.id.11384 | MCP | rs28568391 | 0.733 | 0.961 | 0.564 | 0.434 | 0.804 | 0.634 | 0.450 |
| genus.ErysipelotrichaceaeUCG003.id.11384 | MCP | rs4758231 | 0.515 | 0.080 | 0.485 | 0.095 | 0.783 | 0.999 | 0.660 |
| genus.ErysipelotrichaceaeUCG003.id.11384 | MCP | rs59068084 | 0.426 | 0.202 | 0.979 | 0.032 | 0.584 | 0.350 | 0.919 |
| genus.ErysipelotrichaceaeUCG003.id.11384 | MCP | rs59104037 | 0.577 | 0.135 | 0.592 | 0.118 | 0.712 | 0.685 | 0.540 |
| genus.ErysipelotrichaceaeUCG003.id.11384 | MCP | rs62403464 | 0.011 | 0.391 | 0.321 | 0.125 | 0.010 | 0.338 | 0.723 |
| genus.ErysipelotrichaceaeUCG003.id.11384 | MCP | rs6875357 | 0.179 | 0.063 | 0.049 | 0.717 | 0.481 | 0.240 | 0.569 |
| genus.ErysipelotrichaceaeUCG003.id.11384 | MCP | rs73074432 | 0.259 | 0.061 | 0.814 | 0.437 | 0.086 | 0.073 | 0.331 |
| genus.ErysipelotrichaceaeUCG003.id.11384 | MCP | rs74988980 | 0.285 | 0.262 | 0.975 | 0.530 | 0.336 | 0.284 | 0.781 |
| genus.ErysipelotrichaceaeUCG003.id.11384 | MCP | rs75949021 | 0.750 | 0.189 | 0.525 | 0.304 | 0.312 | 0.551 | 0.511 |
| genus.ErysipelotrichaceaeUCG003.id.11384 | MCP | rs76502207 | 0.496 | 0.169 | 0.863 | 0.074 | 0.116 | 0.296 | 0.240 |
| genus.ErysipelotrichaceaeUCG003.id.11384 | MCP | rs8053479 | 0.606 | 0.427 | 0.428 | 0.178 | 0.228 | 0.949 | 0.412 |
| order.Actinomycetales.id.420 | MCP | rs2889192 | 0.734 | 0.172 | 0.350 | 0.192 | 0.276 | 0.290 | 0.924 |
| order.Actinomycetales.id.420 | MCP | rs34583783 | 0.376 | 0.659 | 0.784 | 0.051 | 0.681 | 0.216 | 0.764 |
| order.Actinomycetales.id.420 | MCP | rs35011108 | 0.704 | 0.896 | 0.692 | 0.942 | 0.290 | 0.730 | 0.644 |
| order.Actinomycetales.id.420 | MCP | rs4073240 | 0.165 | 0.731 | 0.918 | 0.592 | 0.449 | 0.222 | 0.568 |
| order.Actinomycetales.id.420 | MCP | rs58484246 | 0.989 | 0.023 | 0.001 | 0.316 | 0.736 | 0.013 | 0.017 |
| family.Clostridiaceae1.id.1869 | MCP | rs10875374 | 0.217 | 0.618 | 0.777 | 0.787 | 0.382 | 0.800 | 0.343 |
| family.Clostridiaceae1.id.1869 | MCP | rs12186080 | 0.458 | 0.606 | 0.326 | 0.675 | 0.292 | 0.238 | 0.467 |
| family.Clostridiaceae1.id.1869 | MCP | rs12341505 | 0.721 | 0.645 | 0.804 | 0.649 | 0.740 | 0.190 | 0.087 |
| family.Clostridiaceae1.id.1869 | MCP | rs2795528 | 0.524 | 0.121 | 0.167 | 0.962 | 0.159 | 0.720 | 0.342 |
| family.Clostridiaceae1.id.1869 | MCP | rs2817172 | 0.419 | 0.642 | 0.661 | 0.571 | 0.635 | 0.536 | 0.510 |
| family.Clostridiaceae1.id.1869 | MCP | rs4723021 | 0.985 | 0.738 | 0.712 | 0.606 | 0.663 | 0.729 | 0.548 |
| family.Clostridiaceae1.id.1869 | MCP | rs550843 | 0.487 | 0.368 | 0.171 | 0.967 | 0.322 | 0.930 | 0.275 |
| family.Clostridiaceae1.id.1869 | MCP | rs56188186 | 0.402 | 0.355 | 0.480 | 0.275 | 0.569 | 0.707 | 0.506 |
| family.Clostridiaceae1.id.1869 | MCP | rs62397761 | 0.372 | 0.056 | 0.034 | 0.733 | 0.090 | 0.015 | 0.193 |
| family.Clostridiaceae1.id.1869 | MCP | rs881532 | 0.784 | 0.360 | 0.885 | 0.884 | 0.572 | 0.945 | 0.606 |
| order.Gastranaerophilales.id.1591 | MCP | rs11150282 | 0.103 | 0.336 | 0.892 | 0.035 | 0.072 | 0.718 | 0.086 |
| order.Gastranaerophilales.id.1591 | MCP | rs113884518 | 0.316 | 0.905 | 0.991 | 0.199 | 0.515 | 0.717 | 0.411 |
| order.Gastranaerophilales.id.1591 | MCP | rs28678345 | 0.028 | 0.963 | 0.256 | 0.752 | 0.032 | 0.216 | 0.703 |
| order.Gastranaerophilales.id.1591 | MCP | rs367480 | 0.020 | 0.688 | 0.228 | 0.748 | 0.181 | 0.178 | 0.647 |
| order.Gastranaerophilales.id.1591 | MCP | rs4129395 | 0.981 | 0.405 | 0.277 | 0.912 | 0.806 | 0.139 | 0.090 |
| order.Gastranaerophilales.id.1591 | MCP | rs789069 | 0.897 | 0.204 | 0.135 | 0.185 | 0.004 | 0.459 | 0.641 |
| order.Gastranaerophilales.id.1591 | MCP | rs79790072 | 0.864 | 0.036 | 0.671 | 0.756 | 0.133 | 0.640 | 0.490 |
| order.Gastranaerophilales.id.1591 | MCP | rs8028558 | 0.362 | 0.752 | 0.741 | 0.536 | 0.990 | 0.061 | 0.210 |
| order.Gastranaerophilales.id.1591 | MCP | rs9864379 | 0.224 | 0.274 | 0.061 | 0.687 | 0.819 | 0.276 | 0.976 |
| The associations are not significant after the multiple corrections (Genus Adlercreutzia P<0.05/(7×7)=1.02×10^-3^, Genus Allisonella P<0.05/(7×7)=1.02×10^-3^, Genus Dialister P<0.05/(11×7)=6.49×10^-4^, Genus Anaerofilum P<0.05/(9×7)=7.94×10^-4^, Genus Anaerostipes P<0.05/(11×7)=6.94×10^-4^, Genus ChristensenellaceaeR.7group P<0.05/(8×7)=8.92×10^-4^, Genus LachnospiraceaeNC2004group P<0.05/(9×7)=7.94×10^-3^, Family Actinomycetaceae P<0.05/(5×7)=1.42×10^-3^, Genus ErysipelotrichaceaeUCG003 P<0.05/(16×7)=4.46×10^-4^, Order Actinomycetales P<0.05/(5×7)=1.43×10^-3^, Family Clostridiaceae1 P<0.05/(10×7)=7.14×10^-4^, Order.Gastranaerophilales P<0.05/(9×7)=7.94×10^-4^). POU, prescription opioid use; MCP, multisite chronic pain. | | | | | | | | | |

**Table S3. MR Results of gut microbiota on POU/MCP after removing SNPs associated with traits of chronic bowel or intestine disease.**

|  |  |  |  |  |  | **Directional pleiotropy** | | **Cochran Q-test** | | **Rucker's framework** | | **Steiger** |
| --- | --- | --- | --- | --- | --- | --- | --- | --- | --- | --- | --- | --- |
| **Exposure** | **Outcome** | **Method** | **NSNPs** | **OR/Beta (95% CI)** ^a^ | ***P*** | **Egger intercept  (*P*)** | **MRPRESSO  global test RSSobs (*P*)** | **I^2^  statistics** | **Q-statistic  (*P*)** | **Q'-statistic  (*P*)** | **Q–Q'  (*P*)** | ***P*** |
| genus.  Anaerofilum.  id.2053 | POU | IVW | 6 | 0.928 (0.847, 1.017) | 0.109 | 0.035  (0.335) | 4.346  (0.706) | 0.00% | 3.075  (0.688) | 1.874  (0.759) | 1.200  (0.273) | 1.36E-26 |
|  |  | Weighted median | 6 | 0.941 (0.843, 1.051) | 0.280 |  |  |  |  |  |  |  |
|  |  | MR Egger | 6 | 0.682 (0.390, 1.192) | 0.250 |  |  |  |  |  |  |  |
| genus.  Anaerostipes.  id.1991 | POU | IVW | 5 | 0.894 (0.703, 1.136) | 0.358 | 0.039  (0.11) | 11.374  (0.171) | 46.28% | 7.446  (0.114) | 2.376  (0.498) | 5.070  (0.024) | 5.09E-17 |
|  |  | Weighted median | 5 | 0.891 (0.679, 1.170) | 0.407 |  |  |  |  |  |  |  |
|  |  | MR Egger | 5 | 0.545 (0.343, 0.868) | 0.083 |  |  |  |  |  |  |  |
| genus.  ChristensenellaceaeR.  7group.  id.11283 | POU | IVW | 5 | 0.785 (0.656, 0.941) | 0.009 | 0.009  (0.641) | 1.793  (0.926) | 0.00% | 1.121  (0.891) | 0.854  (0.837) | 0.267  (0.605) | 1.83E-16 |
|  |  | Weighted median | 5 | 0.811 (0.641, 1.026) | 0.080 |  |  |  |  |  |  |  |
|  |  | MR Egger | 5 | 0.698 (0.432, 1.129) | 0.239 |  |  |  |  |  |  |  |
| genus.  Lachnospiraceae  NC2004group.  id.11316 | POU | IVW | 7 | 0.894 (0.812, 0.984) | 0.022 | 0.022  (0.439) | 7.046  (0.567) | 0.00% | 5.075  (0.534) | 4.368  (0.498) | 0.707  (0.401) | 3.92E-28 |
|  |  | Weighted median | 7 | 0.864 (0.760, 0.983) | 0.026 |  |  |  |  |  |  |  |
|  |  | MR Egger | 7 | 0.738 (0.468, 1.165) | 0.249 |  |  |  |  |  |  |  |
| genus.  Adlercreutzia.  id.812 | POU | IVW | 5 | 1.140 (0.959, 1.355) | 0.138 | -0.007  (0.871) | 11.245  (0.175) | 43.71% | 7.106  (0.13) | 7.033  (0.071) | 0.074  (0.786) | 9.76E-19 |
|  |  | Weighted median | 5 | 1.072 (0.889, 1.293) | 0.466 |  |  |  |  |  |  |  |
|  |  | MR Egger | 5 | 1.234 (0.502, 3.034) | 0.678 |  |  |  |  |  |  |  |
| genus.  Allisonella.  id.2174 | POU | IVW | 5 | 1.133 (1.048, 1.224) | 0.002 | -0.082  (0.442) | 1.429  (0.95) | 0.00% | 0.893  (0.926) | 0.112  (0.99) | 0.782  (0.377) | 1.38E-23 |
|  |  | Weighted median | 5 | 1.112 (1.009, 1.226) | 0.032 |  |  |  |  |  |  |  |
|  |  | MR Egger | 5 | 1.912 (0.597, 6.123) | 0.355 |  |  |  |  |  |  |  |
| genus.  Dialister.  id.2183 | POU | IVW | 8 | 1.106 (0.975, 1.255) | 0.118 | 0.009  (0.746) | 4.873  (0.833) | 0.00% | 3.593  (0.825) | 3.478  (0.747) | 0.115  (0.735) | 3.71E-29 |
|  |  | Weighted median | 8 | 1.095 (0.931, 1.288) | 0.274 |  |  |  |  |  |  |  |
|  |  | MR Egger | 8 | 0.967 (0.439, 2.129) | 0.935 |  |  |  |  |  |  |  |
| family.  Actinomycetaceae.  id.42 | MCP | IVW | 4 | -0.023(-0.052, 0.005) | 0.113 | 0.004  (0.412) | 2.226  (0.763) | 0.00% | 1.188  (0.756) | 0.130  (0.937) | 1.058  (0.304) | 1.84E-21 |
|  |  | Weighted median | 4 | -0.030(-0.066, 0.006) | 0.098 |  |  |  |  |  |  |  |
|  |  | MR Egger | 4 | -0.057(-0.127, 0.013) | 0.253 |  |  |  |  |  |  |  |
| genus.  Erysipelotrichaceae  UCG003.  id.11384 | MCP | IVW | 9 | -0.019(-0.046, 0.009) | 0.180 | 0.002  (0.543) | 10.110  (0.462) | 3.74% | 8.311  (0.404) | 7.852  (0.346) | 0.459  (0.498) | 8.77E-45 |
|  |  | Weighted median | 9 | -0.035(-0.071, 0.000) | 0.052 |  |  |  |  |  |  |  |
|  |  | MR Egger | 9 | -0.042 (-0.118, 0.034) | 0.317 |  |  |  |  |  |  |  |
| order.  Actinomycetales.  id.420 | MCP | IVW | 4 | -0.023(-0.052, 0.006) | 0.113 | 0.004  (0.412) | 2.235  (0.762) | 0.00% | 1.191  (0.755) | 0.133  (0.936) | 1.059  (0.304) | 1.93E-21 |
|  |  | Weighted median | 4 | -0.030(-0.068, 0.007) | 0.110 |  |  |  |  |  |  |  |
|  |  | MR Egger | 4 | -0.057(-0.128, 0.013) | 0.254 |  |  |  |  |  |  |  |
| family.  Clostridiaceae1.  id.1869 | MCP | IVW | 9 | 0.035 (0.007, 0.062) | 0.013 | 0.001  (0.73) | 8.382  (0.586) | 0.00% | 6.823  (0.556) | 6.693  (0.461) | 0.129  (0.719) | 8.90E-40 |
|  |  | Weighted median | 9 | 0.027 (-0.011, 0.064) | 0.166 |  |  |  |  |  |  |  |
|  |  | MR Egger | 9 | 0.022 (-0.053, 0.097) | 0.583 |  |  |  |  |  |  |  |
| order.  Gastranaerophilales.  id.1591 | MCP | IVW | 4 | 0.008 (-0.018, 0.034) | 0.557 | 0.000  (0.992) | 5.028  (0.454) | 1.74% | 3.053  (0.384) | 3.053  (0.217) | 0.000  (0.989) | 4.41E-21 |
|  |  | Weighted median | 4 | 0.010 (-0.022, 0.041) | 0.551 |  |  |  |  |  |  |  |
|  |  | MR Egger | 4 | 0.008 (-0.085, 0.101) | 0.876 |  |  |  |  |  |  |  |
|  | | | | | | | | | | | | |

**Table S4. MR Results of metabolites on POU.**

|  |  |  |  |  |  | **Directional pleiotropy** | | **Cochran Q-test** | | **Rucker's framework** | | **Steiger** |
| --- | --- | --- | --- | --- | --- | --- | --- | --- | --- | --- | --- | --- |
| **Exposure** | **Outcome** | **Method** | **NSNPs** | **OR (95% CI)** | ***P*** | **Egger intercept  (*P*)** | **MRPRESSO  global test RSSobs (*P*)** | **I^2^  statistics** | **Q-statistic  (*P*)** | **Q'-statistic  (*P*)** | **Q–Q'  (*P*)** | ***P*** |
| 5-hydroxyin  doleacetic acid | POU | IVW | 11 | 0.636 (0.46, 0.878) | 0.006 | 0.001  (0.923) | 8.206  (0.748) | 0.00% | 7.058  (0.72) | 7.049  (0.632) | 0.01  (0.921) | 5.09E-50 |
|  |  | Weighted median | 11 | 0.651 (0.42, 1.009) | 0.055 |  |  |  |  |  |  |  |
|  |  | MR Egger | 11 | 0.617 (0.318, 1.2) | 0.188 |  |  |  |  |  |  |  |
| guanosine diphosphate | POU | IVW | 9 | 0.567 (0.348, 0.923) | 0.023 | -0.004  (0.832) | 16.68  (0.126) | 40.25% | 13.389  (0.099) | 13.296  (0.065) | 0.092  (0.761) | 8.47E-41 |
|  |  | Weighted median | 9 | 0.63 (0.367, 1.081) | 0.093 |  |  |  |  |  |  |  |
|  |  | MR Egger | 9 | 0.689 (0.113, 4.184) | 0.698 |  |  |  |  |  |  |  |
| indoxyl sulfate | POU | IVW | 7 | 0.591 (0.403, 0.865) | 0.007 | 0.004  (0.82) | 7.313  (0.625) | 0.00% | 5.784  (0.448) | 5.718  (0.335) | 0.066  (0.798) | 1.12E-43 |
|  |  | Weighted median | 7 | 0.559 (0.337, 0.927) | 0.024 |  |  |  |  |  |  |  |
|  |  | MR Egger | 7 | 0.503 (0.127, 1.996) | 0.373 |  |  |  |  |  |  |  |
| TAG 50:4 | POU | IVW | 8 | 1.594 (1.025, 2.48) | 0.039 | -0.001  (0.94) | 6.124  (0.813) | 0.00% | 3.874  (0.794) | 3.868  (0.695) | 0.006  (0.938) | 1.30E-47 |
|  |  | Weighted median | 8 | 1.956 (1.121, 3.415) | 0.018 |  |  |  |  |  |  |  |
|  |  | MR Egger | 8 | 1.664 (0.516, 5.373) | 0.427 |  |  |  |  |  |  |  |
| TAG 52:2 | POU | IVW | 12 | 1.57 (1.093, 2.254) | 0.015 | 0  (0.985) | 9.925  (0.724) | 0.00% | 8.177  (0.697) | 8.177  (0.612) | 0.000  (0.985) | 8.71E-59 |
|  |  | Weighted median | 12 | 1.723 (1.053, 2.818) | 0.030 |  |  |  |  |  |  |  |
|  |  | MR Egger | 12 | 1.597 (0.27, 9.454) | 0.617 |  |  |  |  |  |  |  |
| TAG 52:6 | POU | IVW | 7 | 1.424 (1.072, 1.89) | 0.015 | 0.007  (0.455) | 8.97  (0.616) | 0.00% | 3.985  (0.679) | 3.33  (0.649) | 0.656  (0.418) | 2.42E-31 |
|  |  | Weighted median | 7 | 1.39 (0.969, 1.993) | 0.074 |  |  |  |  |  |  |  |
|  |  | MR Egger | 7 | 1.239 (0.798, 1.924) | 0.384 |  |  |  |  |  |  |  |
| TAG 54:2 | POU | IVW | 5 | 1.766 (1.032, 3.02) | 0.038 | -0.002  (0.916) | 1.889  (0.876) | 0.00% | 1.222  (0.874) | 1.209  (0.751) | 0.013  (0.909) | 3.41E-29 |
|  |  | Weighted median | 5 | 1.768 (0.906, 3.449) | 0.095 |  |  |  |  |  |  |  |
|  |  | MR Egger | 5 | 1.973 (0.272, 14.337) | 0.550 |  |  |  |  |  |  |  |
| TAG 58:11 | POU | IVW | 6 | 2.193 (1.377, 3.491) | 0.001 | 0.001  (0.973) | 6.285  (0.551) | 0.00% | 4.646  (0.461) | 4.645  (0.326) | 0.002  (0.969) | 9.76E-38 |
|  |  | Weighted median | 6 | 2.626 (1.412, 4.883) | 0.002 |  |  |  |  |  |  |  |
|  |  | MR Egger | 6 | 2.137 (0.495, 9.222) | 0.366 |  |  |  |  |  |  |  |
| CE 14:0 | POU | IVW | 9 | 0.597 (0.395, 0.902) | 0.014 | 0.012  (0.46) | 5.083  (0.86) | 0.00% | 4.129  (0.845) | 3.517  (0.833) | 0.612  (0.434) | 9.41E-44 |
|  |  | Weighted median | 9 | 0.623 (0.365, 1.064) | 0.083 |  |  |  |  |  |  |  |
|  |  | MR Egger | 9 | 0.344 (0.081, 1.454) | 0.190 |  |  |  |  |  |  |  |
| CE 20:4 | POU | IVW | 13 | 1.347 (1.017, 1.783) | 0.038 | 0.006  (0.567) | 16.737  (0.338) | 9.31% | 13.231  (0.352) | 12.826  (0.305) | 0.405  (0.524) | 1.29E-101 |
|  |  | Weighted median | 13 | 1.55 (1.053, 2.281) | 0.026 |  |  |  |  |  |  |  |
|  |  | MR Egger | 13 | 1.111 (0.552, 2.239) | 0.773 |  |  |  |  |  |  |  |
| DAG 36:2 | POU | IVW | 8 | 1.523 (1.056, 2.197) | 0.024 | 0.005  (0.675) | 4.41  (0.867) | 0.00% | 3.457  (0.84) | 3.263  (0.775) | 0.194  (0.659) | 1.12E-44 |
|  |  | Weighted median | 8 | 1.692 (1.028, 2.784) | 0.038 |  |  |  |  |  |  |  |
|  |  | MR Egger | 8 | 1.325 (0.645, 2.72) | 0.472 |  |  |  |  |  |  |  |
| PC 36:3 | POU | IVW | 7 | 0.693 (0.514, 0.934) | 0.016 | -0.001  (0.879) | 6.041  (0.679) | 0.00% | 5.232  (0.514) | 5.205  (0.391) | 0.027  (0.87) | 2.47E-33 |
|  |  | Weighted median | 7 | 0.702 (0.473, 1.042) | 0.079 |  |  |  |  |  |  |  |
|  |  | MR Egger | 7 | 0.71 (0.466, 1.08) | 0.170 |  |  |  |  |  |  |  |
| MR, mendelian randomization; POU, prescription opioid use; IVW, inverse variance weighted; NSNPs, number of single nucleotide polymorphisms; OR, odds ratio for per 10 units increase in metabolites on POU; CI, confidence interval; RSSobs, residual sums of squares of observations; TAG, triacylglycerol; CE, cholesterol ester; DAG, diacylglycerol; PC, phosphatidylcholine. | | | | | | | | | | | | |

**Table S5. MR Results of metabolites on MCP.**

|  |  |  |  |  |  | **Directional pleiotropy** | | **Cochran Q-test** | | **Rucker's framework** | | **Steiger** |
| --- | --- | --- | --- | --- | --- | --- | --- | --- | --- | --- | --- | --- |
| **Exposure** | **Outcome** | **Method** | **NSNPs** | **Beta (95% CI)** | ***P*** | **Egger intercept  (*P*)** | **MRPRESSO  global test, RSSobs (*P*)** | **I^2^  statistics** | **Q-statistic  (*P*)** | **Q'-statistic  (*P*)** | **Q–Q'  (*P*)** | ***P*** |
| asparagine | MCP | IVW | 9 | 0.131 (0.043, 0.219) | 0.004 | -0.002  (0.5) | 5.944  (0.806) | 0.00% | 4.686  (0.791) | 4.181  (0.759) | 0.506  (0.477) | 5.45E-50 |
|  |  | Weighted median | 9 | 0.15 (0.027, 0.273) | 0.016 |  |  |  |  |  |  |  |
|  |  | MR Egger | 9 | 0.22 (-0.043, 0.483) | 0.143 |  |  |  |  |  |  |  |
| isoleucine | MCP | IVW | 10 | 0.077 (0.010, 0.144) | 0.023 | 0.001  (0.784) | 11.773  (0.428) | 6.34% | 9.61  (0.383) | 9.514  (0.301) | 0.096  (0.757) | 1.42E-52 |
|  |  | Weighted median | 10 | 0.051 (-0.043, 0.145) | 0.290 |  |  |  |  |  |  |  |
|  |  | MR Egger | 10 | 0.064 (-0.050, 0.178) | 0.305 |  |  |  |  |  |  |  |
| PC 40:6 | MCP | IVW | 12 | 0.088 (0.006, 0.170) | 0.038 | -0.003  (0.273) | 15.514  (0.331) | 15.06% | 12.951  (0.297) | 11.412  (0.326) | 1.539  (0.215) | 1.14E-66 |
|  |  | Weighted median | 12 | 0.099 (-0.013, 0.211) | 0.083 |  |  |  |  |  |  |  |
|  |  | MR Egger | 12 | 0.208 (-0.012, 0.428) | 0.092 |  |  |  |  |  |  |  |
| TAG 46:1 | MCP | IVW | 10 | 0.114 (0.022, 0.206) | 0.016 | -0.005  (0.29) | 10.53  (0.529) | 0.00% | 8.629  (0.472) | 7.342  (0.5) | 1.286  (0.257) | 8.39E-52 |
|  |  | Weighted median | 10 | 0.092 (-0.031, 0.215) | 0.146 |  |  |  |  |  |  |  |
|  |  | MR Egger | 10 | 0.362 (-0.077, 0.801) | 0.144 |  |  |  |  |  |  |  |
| TAG 48:3 | MCP | IVW | 11 | 0.073 (0.020, 0.126) | 0.006 | 0.003  (0.196) | 9.443  (0.681) | 0.00% | 8.263  (0.603) | 6.316  (0.708) | 1.947  (0.163) | 6.83E-59 |
|  |  | Weighted median | 11 | 0.069 (-0.013, 0.151) | 0.099 |  |  |  |  |  |  |  |
|  |  | MR Egger | 11 | 0.013 (-0.087, 0.113) | 0.804 |  |  |  |  |  |  |  |
| TAG 50:3 | MCP | IVW | 16 | 0.128 (0.034, 0.222) | 0.008 | 0.002  (0.5) | 11.892  (0.499) | 0.00% | 8.687  (0.467) | 7.637  (0.459) | 1.05  (0.305) | 1.45E-82 |
|  |  | Weighted median | 16 | 0.063 (-0.041, 0.167) | 0.230 |  |  |  |  |  |  |  |
|  |  | MR Egger | 16 | 0.045 (-0.210, 0.300) | 0.736 |  |  |  |  |  |  |  |
| TAG 52:1 | MCP | IVW | 10 | 0.168 (0.074, 0.262) | 0.000 | -0.007  (0.337) | 6.017  (0.859) | 0.00% | 4.937  (0.84) | 3.894  (0.867) | 1.043  (0.307) | 3.08E-53 |
|  |  | Weighted median | 10 | 0.158 (0.036, 0.280) | 0.011 |  |  |  |  |  |  |  |
|  |  | MR Egger | 10 | 0.513 (-0.155, 1.181) | 0.171 |  |  |  |  |  |  |  |
| xanthine | MCP | IVW | 8 | 0.065 (0.018, 0.112) | 0.007 | 0.002  (0.153) | 14.776  (0.494) | 17.77% | 8.512  (0.29) | 5.838  (0.442) | 2.674  (0.102) | 2.77E-39 |
|  |  | Weighted median | 8 | 0.056 (-0.013, 0.125) | 0.109 |  |  |  |  |  |  |  |
|  |  | MR Egger | 8 | 0.042 (-0.009, 0.093) | 0.161 |  |  |  |  |  |  |  |
| cyclic adenosine monophosphate | MCP | IVW | 8 | -0.116 (-0.204, -0.028) | 0.010 | 0.004  (0.517) | 10.643  (0.371) | 18.41% | 8.58  (0.284) | 7.953  (0.242) | 0.627  (0.429) | 5.10E-40 |
|  |  | Weighted median | 8 | -0.117 (-0.229, -0.005) | 0.039 |  |  |  |  |  |  |  |
|  |  | MR Egger | 8 | -0.282 (-0.762, 0.198) | 0.294 |  |  |  |  |  |  |  |
| inositol | MCP | IVW | 7 | -0.12 (-0.204, -0.036) | 0.005 | 0.003  (0.402) | 10.836  (0.364) | 0.00% | 5.65  (0.463) | 4.813  (0.439) | 0.838  (0.36) | 4.54E-34 |
|  |  | Weighted median | 7 | -0.065 (-0.185, 0.055) | 0.288 |  |  |  |  |  |  |  |
|  |  | MR Egger | 7 | -0.193 (-0.371, -0.015) | 0.086 |  |  |  |  |  |  |  |
| SM 24:0 | MCP | IVW | 6 | -0.113 (-0.213, -0.013) | 0.025 | 0.003  (0.419) | 7.21  (0.509) | 0.00% | 4.291  (0.508) | 3.481  (0.481) | 0.81  (0.368) | 1.47E-29 |
|  |  | Weighted median | 6 | -0.143 (-0.274, -0.012) | 0.031 |  |  |  |  |  |  |  |
|  |  | MR Egger | 6 | -0.186 (-0.372, 0.000) | 0.122 |  |  |  |  |  |  |  |
| TAG 56:10 | MCP | IVW | 4 | -0.146 (-0.268, -0.024) | 0.020 | 0.002  (0.722) | 0.751  (0.934) | 0.00% | 0.435  (0.933) | 0.267  (0.875) | 0.167  (0.683) | 2.46E-23 |
|  |  | Weighted median | 4 | -0.159 (-0.308, -0.010) | 0.036 |  |  |  |  |  |  |  |
|  |  | MR Egger | 4 | -0.209 (-0.536, 0.118) | 0.337 |  |  |  |  |  |  |  |
| MR, mendelian randomization; MCP, multisite chronic pain; IVW, inverse-variance weighted; NSNPs, number of single nucleotide polymorphisms; Beta, MR effect estimate for per 10 units increase in metabolites on MCP; CI, confidence interval; RSSobs, residual sums of squares of observations; PC, phosphatidylcholine; TAG, triacylglycerol; SM, sphingomyelin. | | | | | | | | | | | | |

**Table S6. MR Power calculation for detecting significant (*P* < 0.05) causal effect (OR = 1.2) of gut microbiota on the risk of POU, and (beta = 0.1) of gut microbiota on MCP.**

|  | **POU** | | | **MCP** | | |
| --- | --- | --- | --- | --- | --- | --- |
| **Gut microbiota** | **NSNPs** | **R^2^** | **Power** | **NSNPs** | **R^2^** | **Power** |
| class.Actinobacteria.id.419 | 13 | 0.022 | 0.95 | 15 | 0.030 | 1.00 |
| class.Alphaproteobacteria.id.2379 | 6 | 0.021 | 0.94 | 7 | 0.023 | 1.00 |
| class.Bacilli.id.1673 | 17 | 0.034 | 0.99 | 18 | 0.036 | 1.00 |
| class.Bacteroidia.id.912 | 12 | 0.022 | 0.95 | 14 | 0.031 | 1.00 |
| class.Betaproteobacteria.id.2867 | 12 | 0.020 | 0.92 | 12 | 0.020 | 1.00 |
| class.Clostridia.id.1859 | 12 | 0.020 | 0.93 | 12 | 0.020 | 1.00 |
| class.Coriobacteriia.id.809 | 10 | 0.021 | 0.94 | 14 | 0.026 | 1.00 |
| class.Deltaproteobacteria.id.3087 | 13 | 0.022 | 0.95 | 13 | 0.022 | 1.00 |
| class.Erysipelotrichia.id.2147 | 11 | 0.014 | 0.81 | 13 | 0.016 | 0.94 |
| class.Gammaproteobacteria.id.3303 | 7 | 0.014 | 0.80 | 7 | 0.014 | 0.93 |
| class.Lentisphaeria.id.2250 | 7 | 0.045 | 1.00 | 8 | 0.049 | 1.00 |
| class.Melainabacteria.id.1589 | 10 | 0.053 | 1.00 | 10 | 0.053 | 1.00 |
| class.Methanobacteria.id.119 | 9 | 0.068 | 1.00 | 10 | 0.074 | 1.00 |
| class.Mollicutes.id.3920 | 11 | 0.021 | 0.94 | 12 | 0.026 | 1.00 |
| class.Negativicutes.id.2164 | 11 | 0.016 | 0.87 | 12 | 0.017 | 0.95 |
| class.Verrucomicrobiae.id.4029 | 11 | 0.023 | 0.96 | 11 | 0.025 | 1.00 |
| family.Acidaminococcaceae.id.2166 | 7 | 0.016 | 0.87 | 7 | 0.016 | 0.94 |
| family.Actinomycetaceae.id.421 | 5 | 0.017 | 0.88 | 5 | 0.017 | 0.95 |
| family.Alcaligenaceae.id.2875 | 12 | 0.023 | 0.95 | 12 | 0.024 | 1.00 |
| family.Bacteroidaceae.id.917 | 8 | 0.013 | 0.77 | 9 | 0.015 | 0.93 |
| family.BacteroidalesS24.7group.id.11173 | 8 | 0.030 | 0.99 | 9 | 0.033 | 1.00 |
| family.Bifidobacteriaceae.id.433 | 8 | 0.020 | 0.93 | 12 | 0.031 | 1.00 |
| family.Christensenellaceae.id.1866 | 11 | 0.026 | 0.98 | 11 | 0.026 | 1.00 |
| family.Clostridiaceae1.id.1869 | 10 | 0.018 | 0.90 | 10 | 0.018 | 0.96 |
| family.ClostridialesvadinBB60group.id.11286 | 15 | 0.040 | 1.00 | 15 | 0.040 | 1.00 |
| family.Coriobacteriaceae.id.811 | 10 | 0.021 | 0.94 | 14 | 0.026 | 1.00 |
| family.Defluviitaleaceae.id.1924 | 9 | 0.028 | 0.98 | 11 | 0.033 | 1.00 |
| family.Desulfovibrionaceae.id.3169 | 10 | 0.020 | 0.92 | 10 | 0.021 | 1.00 |
| family.Enterobacteriaceae.id.3469 | 6 | 0.014 | 0.82 | 7 | 0.017 | 0.95 |
| family.Erysipelotrichaceae.id.2149 | 11 | 0.014 | 0.81 | 13 | 0.016 | 0.94 |
| family.FamilyXI.id.1936 | 8 | 0.069 | 1.00 | 8 | 0.069 | 1.00 |
| family.FamilyXIII.id.1957 | 8 | 0.013 | 0.77 | 11 | 0.021 | 1.00 |
| family.Lachnospiraceae.id.1987 | 15 | 0.025 | 0.97 | 17 | 0.027 | 1.00 |
| family.Lactobacillaceae.id.1836 | 8 | 0.040 | 1.00 | 9 | 0.043 | 1.00 |
| family.Methanobacteriaceae.id.121 | 9 | 0.068 | 1.00 | 10 | 0.074 | 1.00 |
| family.Oxalobacteraceae.id.2966 | 12 | 0.059 | 1.00 | 14 | 0.067 | 1.00 |
| family.Pasteurellaceae.id.3689 | 13 | 0.057 | 1.00 | 14 | 0.059 | 1.00 |
| family.Peptococcaceae.id.2024 | 8 | 0.023 | 0.95 | 9 | 0.025 | 1.00 |
| family.Peptostreptococcaceae.id.2042 | 12 | 0.027 | 0.98 | 14 | 0.031 | 1.00 |
| family.Porphyromonadaceae.id.943 | 8 | 0.012 | 0.77 | 9 | 0.013 | 0.90 |
| family.Prevotellaceae.id.960 | 15 | 0.032 | 0.99 | 16 | 0.034 | 1.00 |
| family.Rhodospirillaceae.id.2717 | 14 | 0.041 | 1.00 | 15 | 0.043 | 1.00 |
| family.Rikenellaceae.id.967 | 16 | 0.024 | 0.97 | 19 | 0.029 | 1.00 |
| family.Ruminococcaceae.id.2050 | 10 | 0.023 | 0.95 | 10 | 0.023 | 1.00 |
| family.Streptococcaceae.id.1850 | 14 | 0.025 | 0.97 | 14 | 0.025 | 1.00 |
| family.unknownfamily.id.1000001214 | 9 | 0.049 | 1.00 | 9 | 0.049 | 1.00 |
| family.unknownfamily.id.1000005471 | 12 | 0.028 | 0.98 | 13 | 0.036 | 1.00 |
| family.unknownfamily.id.1000006161 | 13 | 0.071 | 1.00 | 13 | 0.076 | 1.00 |
| family.Veillonellaceae.id.2172 | 18 | 0.040 | 1.00 | 19 | 0.041 | 1.00 |
| family.Verrucomicrobiaceae.id.4036 | 11 | 0.023 | 0.96 | 11 | 0.025 | 1.00 |
| family.Victivallaceae.id.2255 | 11 | 0.080 | 1.00 | 12 | 0.086 | 1.00 |
| genus.Clostridiuminnocuumgroup.id.14397 | 7 | 0.045 | 1.00 | 9 | 0.056 | 1.00 |
| genus.Eubacteriumbrachygroup.id.11296 | 10 | 0.061 | 1.00 | 10 | 0.061 | 1.00 |
| genus.Eubacteriumcoprostanoligenesgroup.id.11375 | 11 | 0.016 | 0.86 | 13 | 0.020 | 0.99 |
| genus.Eubacteriumeligensgroup.id.14372 | 7 | 0.021 | 0.94 | 7 | 0.021 | 1.00 |
| genus.Eubacteriumfissicatenagroup.id.14373 | 7 | 0.040 | 1.00 | 9 | 0.052 | 1.00 |
| genus.Eubacteriumhalliigroup.id.11338 | 16 | 0.034 | 0.99 | 16 | 0.034 | 1.00 |
| genus.Eubacteriumnodatumgroup.id.11297 | 11 | 0.072 | 1.00 | 11 | 0.072 | 1.00 |
| genus.Eubacteriumoxidoreducensgroup.id.11339 | 4 | 0.017 | 0.89 | 5 | 0.025 | 1.00 |
| genus.Eubacteriumrectalegroup.id.14374 | 8 | 0.017 | 0.89 | 8 | 0.017 | 0.95 |
| genus.Eubacteriumruminantiumgroup.id.11340 | 17 | 0.055 | 1.00 | 18 | 0.057 | 1.00 |
| genus.Eubacteriumventriosumgroup.id.11341 | 12 | 0.019 | 0.92 | 15 | 0.025 | 1.00 |
| genus.Eubacteriumxylanophilumgroup.id.14375 | 7 | 0.018 | 0.89 | 9 | 0.021 | 1.00 |
| genus.Ruminococcusgauvreauiigroup.id.11342 | 10 | 0.018 | 0.90 | 12 | 0.022 | 1.00 |
| genus.Ruminococcusgnavusgroup.id.14376 | 12 | 0.043 | 1.00 | 12 | 0.043 | 1.00 |
| genus.Ruminococcustorquesgroup.id.14377 | 9 | 0.023 | 0.96 | 9 | 0.023 | 1.00 |
| genus.Actinomyces.id.423 | 7 | 0.026 | 0.97 | 7 | 0.026 | 1.00 |
| genus.Adlercreutzia.id.812 | 7 | 0.029 | 0.98 | 8 | 0.031 | 1.00 |
| genus.Akkermansia.id.4037 | 11 | 0.023 | 0.96 | 11 | 0.025 | 1.00 |
| genus.Alistipes.id.968 | 13 | 0.017 | 0.88 | 14 | 0.018 | 0.99 |
| genus.Allisonella.id.2174 | 7 | 0.055 | 1.00 | 8 | 0.062 | 1.00 |
| genus.Alloprevotella.id.961 | 6 | 0.048 | 1.00 | 6 | 0.048 | 1.00 |
| genus.Anaerofilum.id.2053 | 9 | 0.041 | 1.00 | 11 | 0.054 | 1.00 |
| genus.Anaerostipes.id.1991 | 11 | 0.023 | 0.96 | 13 | 0.026 | 1.00 |
| genus.Anaerotruncus.id.2054 | 12 | 0.021 | 0.94 | 13 | 0.024 | 1.00 |
| genus.Bacteroides.id.918 | 8 | 0.013 | 0.77 | 9 | 0.015 | 0.93 |
| genus.Barnesiella.id.944 | 12 | 0.025 | 0.97 | 14 | 0.028 | 1.00 |
| genus.Bifidobacterium.id.436 | 11 | 0.024 | 0.97 | 13 | 0.032 | 1.00 |
| genus.Bilophila.id.3170 | 11 | 0.026 | 0.97 | 13 | 0.029 | 1.00 |
| genus.Blautia.id.1992 | 10 | 0.015 | 0.85 | 13 | 0.022 | 1.00 |
| genus.Butyricicoccus.id.2055 | 8 | 0.020 | 0.93 | 8 | 0.020 | 0.99 |
| genus.Butyricimonas.id.945 | 13 | 0.034 | 0.99 | 13 | 0.036 | 1.00 |
| genus.Butyrivibrio.id.1993 | 14 | 0.093 | 1.00 | 15 | 0.101 | 1.00 |
| genus.CandidatusSoleaferrea.id.11350 | 9 | 0.051 | 1.00 | 10 | 0.058 | 1.00 |
| genus.Catenibacterium.id.2153 | 5 | 0.033 | 0.99 | 5 | 0.033 | 1.00 |
| genus.ChristensenellaceaeR.7group.id.11283 | 8 | 0.013 | 0.79 | 10 | 0.016 | 0.94 |
| genus.Clostridiumsensustricto1.id.1873 | 7 | 0.020 | 0.93 | 7 | 0.020 | 0.99 |
| genus.Collinsella.id.815 | 9 | 0.018 | 0.90 | 9 | 0.018 | 0.98 |
| genus.Coprobacter.id.949 | 10 | 0.049 | 1.00 | 11 | 0.053 | 1.00 |
| genus.Coprococcus1.id.11301 | 12 | 0.024 | 0.96 | 12 | 0.024 | 1.00 |
| genus.Coprococcus2.id.11302 | 8 | 0.019 | 0.92 | 8 | 0.019 | 0.99 |
| genus.Coprococcus3.id.11303 | 8 | 0.013 | 0.80 | 9 | 0.015 | 1.00 |
| genus.DefluviitaleaceaeUCG011.id.11287 | 7 | 0.023 | 0.95 | 9 | 0.029 | 1.00 |
| genus.Desulfovibrio.id.3173 | 8 | 0.021 | 0.94 | 10 | 0.029 | 1.00 |
| genus.Dialister.id.2183 | 11 | 0.022 | 0.95 | 11 | 0.022 | 1.00 |
| genus.Dorea.id.1997 | 9 | 0.017 | 0.89 | 10 | 0.018 | 0.98 |
| genus.Eggerthella.id.819 | 8 | 0.033 | 0.99 | 11 | 0.045 | 1.00 |
| genus.Eisenbergiella.id.11304 | 11 | 0.045 | 1.00 | 11 | 0.045 | 1.00 |
| genus.Enterorhabdus.id.820 | 6 | 0.032 | 0.99 | 7 | 0.035 | 1.00 |
| genus.Erysipelatoclostridium.id.11381 | 13 | 0.031 | 0.99 | 15 | 0.038 | 1.00 |
| genus.ErysipelotrichaceaeUCG003.id.11384 | 15 | 0.030 | 0.99 | 16 | 0.033 | 1.00 |
| genus.Escherichia.Shigella.id.3504 | 9 | 0.028 | 0.98 | 10 | 0.029 | 1.00 |
| genus.Faecalibacterium.id.2057 | 10 | 0.026 | 0.98 | 10 | 0.026 | 1.00 |
| genus.FamilyXIIIAD3011group.id.11293 | 11 | 0.021 | 0.94 | 13 | 0.024 | 1.00 |
| genus.FamilyXIIIUCG001.id.11294 | 8 | 0.020 | 0.93 | 8 | 0.020 | 0.99 |
| genus.Flavonifractor.id.2059 | 5 | 0.017 | 0.89 | 5 | 0.017 | 0.95 |
| genus.Fusicatenibacter.id.11305 | 15 | 0.021 | 0.94 | 18 | 0.025 | 1.00 |
| genus.Gordonibacter.id.821 | 11 | 0.083 | 1.00 | 12 | 0.089 | 1.00 |
| genus.Haemophilus.id.3698 | 9 | 0.046 | 1.00 | 9 | 0.046 | 1.00 |
| genus.Holdemanella.id.11393 | 9 | 0.037 | 1.00 | 11 | 0.047 | 1.00 |
| genus.Holdemania.id.2157 | 14 | 0.046 | 1.00 | 15 | 0.049 | 1.00 |
| genus.Howardella.id.2000 | 9 | 0.058 | 1.00 | 10 | 0.065 | 1.00 |
| genus.Hungatella.id.11306 | 5 | 0.026 | 0.97 | 5 | 0.026 | 1.00 |
| genus.Intestinibacter.id.11345 | 14 | 0.027 | 0.98 | 15 | 0.029 | 1.00 |
| genus.Intestinimonas.id.2062 | 16 | 0.050 | 1.00 | 17 | 0.052 | 1.00 |
| genus.Lachnoclostridium.id.11308 | 13 | 0.018 | 0.90 | 13 | 0.019 | 0.99 |
| genus.Lachnospira.id.2004 | 6 | 0.009 | 0.65 | 6 | 0.009 | 0.86 |
| genus.LachnospiraceaeFCS020group.id.11314 | 12 | 0.033 | 0.99 | 12 | 0.033 | 1.00 |
| genus.LachnospiraceaeNC2004group.id.11316 | 9 | 0.036 | 1.00 | 9 | 0.036 | 1.00 |
| genus.LachnospiraceaeND3007group.id.11317 | 2 | 0.003 | 0.25 | 3 | 0.006 | 0.76 |
| genus.LachnospiraceaeNK4A136group.id.11319 | 14 | 0.028 | 0.98 | 15 | 0.029 | 1.00 |
| genus.LachnospiraceaeUCG001.id.11321 | 11 | 0.032 | 0.99 | 13 | 0.036 | 1.00 |
| genus.LachnospiraceaeUCG004.id.11324 | 12 | 0.020 | 0.93 | 12 | 0.020 | 0.99 |
| genus.LachnospiraceaeUCG008.id.11328 | 11 | 0.041 | 1.00 | 11 | 0.041 | 1.00 |
| genus.LachnospiraceaeUCG010.id.11330 | 8 | 0.020 | 0.93 | 10 | 0.023 | 1.00 |
| genus.Lactobacillus.id.1837 | 8 | 0.035 | 1.00 | 10 | 0.042 | 1.00 |
| genus.Lactococcus.id.1851 | 9 | 0.064 | 1.00 | 9 | 0.064 | 1.00 |
| genus.Marvinbryantia.id.2005 | 9 | 0.019 | 0.91 | 10 | 0.023 | 1.00 |
| genus.Methanobrevibacter.id.123 | 6 | 0.047 | 1.00 | 6 | 0.047 | 1.00 |
| genus.Odoribacter.id.952 | 7 | 0.013 | 0.78 | 7 | 0.014 | 0.93 |
| genus.Olsenella.id.822 | 10 | 0.059 | 1.00 | 11 | 0.064 | 1.00 |
| genus.Oscillibacter.id.2063 | 14 | 0.046 | 1.00 | 14 | 0.046 | 1.00 |
| genus.Oscillospira.id.2064 | 8 | 0.020 | 0.93 | 8 | 0.020 | 0.99 |
| genus.Oxalobacter.id.2978 | 11 | 0.063 | 1.00 | 11 | 0.063 | 1.00 |
| genus.Parabacteroides.id.954 | 6 | 0.019 | 0.91 | 6 | 0.019 | 0.99 |
| genus.Paraprevotella.id.962 | 13 | 0.045 | 1.00 | 13 | 0.045 | 1.00 |
| genus.Parasutterella.id.2892 | 14 | 0.036 | 1.00 | 15 | 0.038 | 1.00 |
| genus.Peptococcus.id.2037 | 11 | 0.064 | 1.00 | 12 | 0.068 | 1.00 |
| genus.Phascolarctobacterium.id.2168 | 9 | 0.024 | 0.96 | 10 | 0.025 | 1.00 |
| genus.Prevotella7.id.11182 | 9 | 0.061 | 1.00 | 11 | 0.074 | 1.00 |
| genus.Prevotella9.id.11183 | 13 | 0.044 | 1.00 | 15 | 0.050 | 1.00 |
| genus.RikenellaceaeRC9gutgroup.id.11191 | 11 | 0.077 | 1.00 | 11 | 0.083 | 1.00 |
| genus.Romboutsia.id.11347 | 13 | 0.029 | 0.98 | 13 | 0.029 | 1.00 |
| genus.Roseburia.id.2012 | 14 | 0.024 | 0.97 | 14 | 0.024 | 1.00 |
| genus.Ruminiclostridium5.id.11355 | 9 | 0.019 | 0.91 | 11 | 0.023 | 1.00 |
| genus.Ruminiclostridium6.id.11356 | 14 | 0.031 | 0.99 | 16 | 0.034 | 1.00 |
| genus.Ruminiclostridium9.id.11357 | 9 | 0.018 | 0.90 | 9 | 0.020 | 0.99 |
| genus.RuminococcaceaeNK4A214group.id.11358 | 12 | 0.024 | 0.97 | 13 | 0.026 | 1.00 |
| genus.RuminococcaceaeUCG002.id.11360 | 20 | 0.038 | 1.00 | 22 | 0.041 | 1.00 |
| genus.RuminococcaceaeUCG003.id.11361 | 12 | 0.023 | 0.95 | 12 | 0.024 | 1.00 |
| genus.RuminococcaceaeUCG004.id.11362 | 8 | 0.021 | 0.94 | 11 | 0.028 | 1.00 |
| genus.RuminococcaceaeUCG005.id.11363 | 14 | 0.029 | 0.99 | 14 | 0.029 | 1.00 |
| genus.RuminococcaceaeUCG009.id.11366 | 11 | 0.035 | 0.99 | 12 | 0.042 | 1.00 |
| genus.RuminococcaceaeUCG010.id.11367 | 6 | 0.013 | 0.79 | 6 | 0.015 | 0.93 |
| genus.RuminococcaceaeUCG011.id.11368 | 8 | 0.050 | 1.00 | 8 | 0.050 | 1.00 |
| genus.RuminococcaceaeUCG013.id.11370 | 12 | 0.023 | 0.95 | 12 | 0.023 | 1.00 |
| genus.RuminococcaceaeUCG014.id.11371 | 10 | 0.029 | 0.99 | 11 | 0.030 | 1.00 |
| genus.Ruminococcus1.id.11373 | 8 | 0.018 | 0.90 | 10 | 0.022 | 1.00 |
| genus.Ruminococcus2.id.11374 | 13 | 0.026 | 0.98 | 15 | 0.029 | 1.00 |
| genus.Sellimonas.id.14369 | 8 | 0.069 | 1.00 | 9 | 0.077 | 1.00 |
| genus.Senegalimassilia.id.11160 | 4 | 0.022 | 0.95 | 5 | 0.025 | 1.00 |
| genus.Slackia.id.825 | 5 | 0.029 | 0.98 | 6 | 0.032 | 1.00 |
| genus.Streptococcus.id.1853 | 14 | 0.022 | 0.95 | 15 | 0.024 | 1.00 |
| genus.Subdoligranulum.id.2070 | 10 | 0.021 | 0.94 | 11 | 0.023 | 1.00 |
| genus.Sutterella.id.2896 | 12 | 0.020 | 0.92 | 12 | 0.020 | 0.99 |
| genus.Terrisporobacter.id.11348 | 5 | 0.022 | 0.94 | 5 | 0.022 | 1.00 |
| genus.Turicibacter.id.2162 | 10 | 0.033 | 0.99 | 10 | 0.035 | 1.00 |
| genus.Tyzzerella3.id.11335 | 12 | 0.055 | 1.00 | 13 | 0.065 | 1.00 |
| genus.unknowngenus.id.1000000073 | 15 | 0.040 | 1.00 | 15 | 0.040 | 1.00 |
| genus.unknowngenus.id.1000001215 | 9 | 0.049 | 1.00 | 9 | 0.049 | 1.00 |
| genus.unknowngenus.id.1000005472 | 12 | 0.028 | 0.98 | 13 | 0.036 | 1.00 |
| genus.unknowngenus.id.1000005479 | 8 | 0.030 | 0.99 | 9 | 0.033 | 1.00 |
| genus.unknowngenus.id.1000006162 | 13 | 0.071 | 1.00 | 13 | 0.076 | 1.00 |
| genus.unknowngenus.id.1868 | 12 | 0.033 | 0.99 | 12 | 0.033 | 1.00 |
| genus.unknowngenus.id.2001 | 10 | 0.028 | 0.98 | 10 | 0.028 | 1.00 |
| genus.unknowngenus.id.2041 | 9 | 0.029 | 0.99 | 12 | 0.037 | 1.00 |
| genus.unknowngenus.id.2071 | 13 | 0.027 | 0.98 | 16 | 0.032 | 1.00 |
| genus.unknowngenus.id.2755 | 12 | 0.037 | 1.00 | 13 | 0.039 | 1.00 |
| genus.unknowngenus.id.826 | 12 | 0.027 | 0.98 | 14 | 0.031 | 1.00 |
| genus.unknowngenus.id.959 | 10 | 0.060 | 1.00 | 12 | 0.070 | 1.00 |
| genus.Veillonella.id.2198 | 5 | 0.019 | 0.91 | 8 | 0.027 | 1.00 |
| genus.Victivallis.id.2256 | 10 | 0.079 | 1.00 | 10 | 0.079 | 1.00 |
| order.Actinomycetales.id.420 | 5 | 0.017 | 0.88 | 5 | 0.017 | 0.95 |
| order.Bacillales.id.1674 | 9 | 0.065 | 1.00 | 9 | 0.074 | 1.00 |
| order.Bacteroidales.id.913 | 12 | 0.022 | 0.95 | 14 | 0.031 | 1.00 |
| order.Bifidobacteriales.id.432 | 8 | 0.020 | 0.93 | 12 | 0.031 | 1.00 |
| order.Burkholderiales.id.2874 | 11 | 0.017 | 0.89 | 11 | 0.019 | 0.99 |
| order.Clostridiales.id.1863 | 13 | 0.020 | 0.93 | 13 | 0.020 | 0.99 |
| order.Coriobacteriales.id.810 | 10 | 0.021 | 0.94 | 14 | 0.026 | 1.00 |
| order.Desulfovibrionales.id.3156 | 12 | 0.021 | 0.94 | 12 | 0.022 | 1.00 |
| order.Enterobacteriales.id.3468 | 6 | 0.014 | 0.82 | 7 | 0.017 | 0.95 |
| order.Erysipelotrichales.id.2148 | 11 | 0.014 | 0.81 | 13 | 0.016 | 0.94 |
| order.Gastranaerophilales.id.1591 | 9 | 0.049 | 1.00 | 9 | 0.049 | 1.00 |
| order.Lactobacillales.id.1800 | 14 | 0.030 | 0.99 | 15 | 0.032 | 1.00 |
| order.Methanobacteriales.id.120 | 9 | 0.068 | 1.00 | 10 | 0.074 | 1.00 |
| order.MollicutesRF9.id.11579 | 12 | 0.028 | 0.98 | 13 | 0.036 | 1.00 |
| order.NB1n.id.3953 | 13 | 0.071 | 1.00 | 13 | 0.076 | 1.00 |
| order.Pasteurellales.id.3688 | 13 | 0.057 | 1.00 | 14 | 0.059 | 1.00 |
| order.Rhodospirillales.id.2667 | 13 | 0.036 | 1.00 | 14 | 0.038 | 1.00 |
| order.Selenomonadales.id.2165 | 11 | 0.016 | 0.87 | 12 | 0.017 | 0.95 |
| order.Verrucomicrobiales.id.4030 | 11 | 0.023 | 0.96 | 11 | 0.025 | 1.00 |
| order.Victivallales.id.2254 | 7 | 0.045 | 1.00 | 8 | 0.049 | 1.00 |
| phylum.Actinobacteria.id.400 | 13 | 0.020 | 0.93 | 15 | 0.025 | 1.00 |
| phylum.Bacteroidetes.id.905 | 10 | 0.020 | 0.93 | 12 | 0.029 | 1.00 |
| phylum.Cyanobacteria.id.1500 | 8 | 0.037 | 1.00 | 8 | 0.037 | 1.00 |
| phylum.Euryarchaeota.id.55 | 11 | 0.068 | 1.00 | 12 | 0.074 | 1.00 |
| phylum.Firmicutes.id.1672 | 14 | 0.025 | 0.97 | 16 | 0.027 | 1.00 |
| phylum.Lentisphaerae.id.2238 | 8 | 0.049 | 1.00 | 9 | 0.054 | 1.00 |
| phylum.Proteobacteria.id.2375 | 11 | 0.017 | 0.88 | 12 | 0.018 | 1.00 |
| phylum.Tenericutes.id.3919 | 11 | 0.021 | 0.94 | 12 | 0.026 | 1.00 |
| phylum.Verrucomicrobia.id.3982 | 11 | 0.026 | 0.98 | 12 | 0.028 | 1.00 |
| MR, mendelian randomization; POU, prescription opioid use; MCP, multisite chronic pain; NSNPs, number of single nucleotide polymorphisms; R^2^, the proportion of variance explained by exposure. R^2^ for each SNP: R^2^ = [beta.exposure^2^]/[se.exposure^2^*N + beta.exposure^2^ ]; R^2^ combined= SUM[R^2^]. | | | | | | |


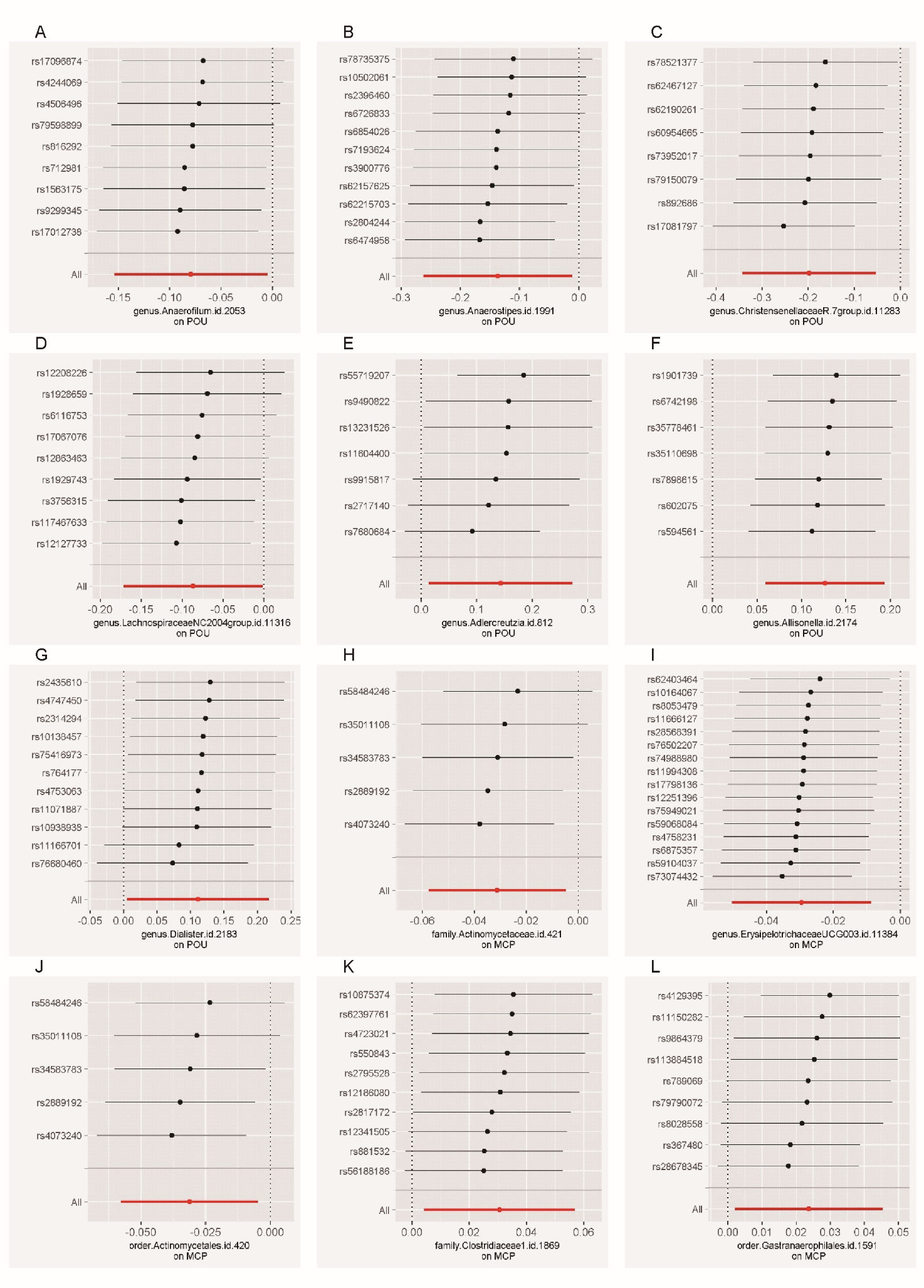


**Figure S1. Leave-one-out analysis of inverse-variance weighted (IVW) estimates between genetically determined gut microbiota traits (*P* < 1 × 10^-5^) and prescription opioid use (POU), as well as multisite chronic pain (MCP).**

Black dots indicated the IVW estimates (raw beta) after leaving a single SNP in turns. Red dots indicated the pooled IVW estimate (raw beta). Horizontal lines indicated the range of 95% confidence interval.

**
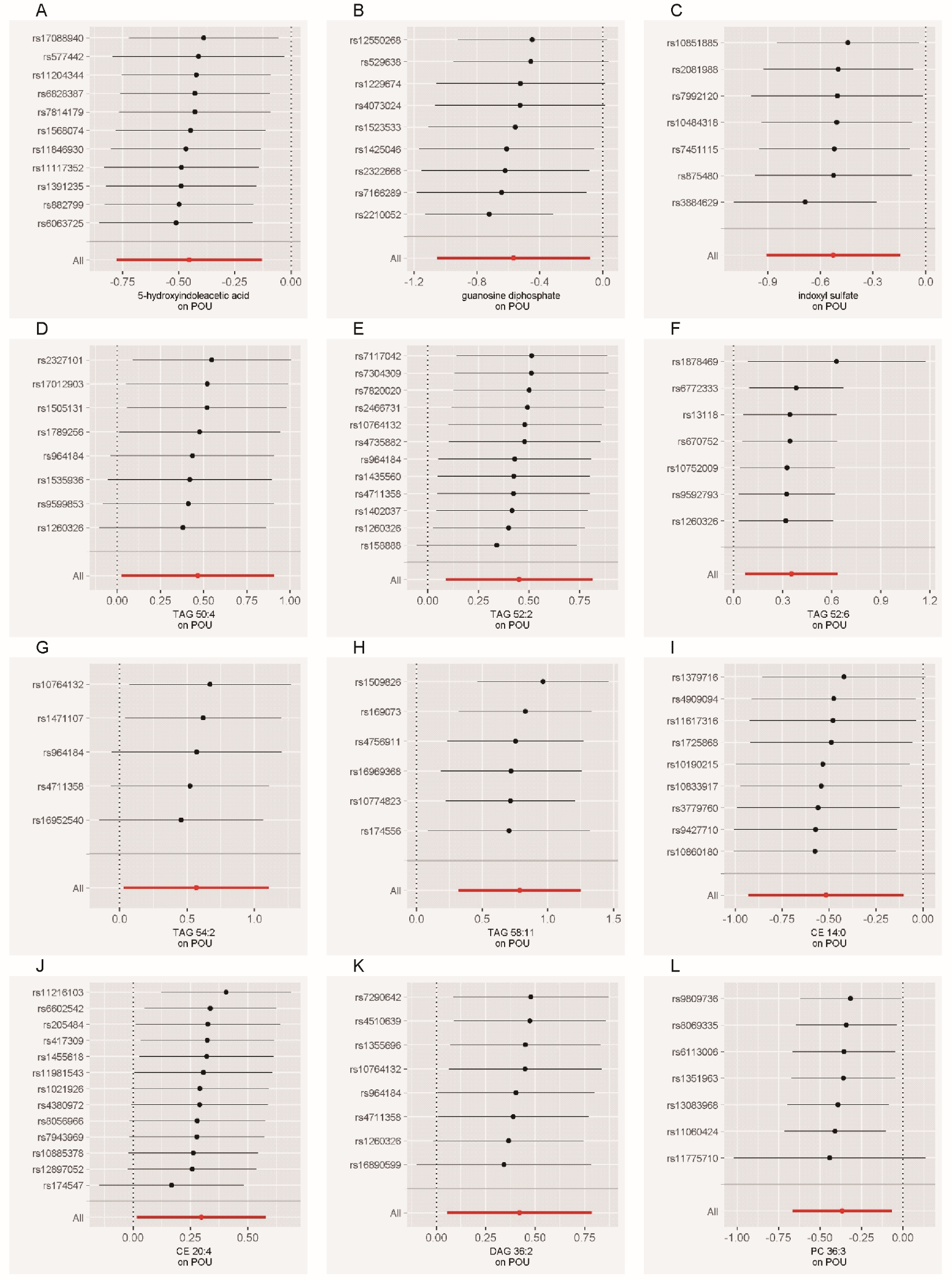
**

**Figure S2. Leave-one-out analysis of inverse-variance weighted (IVW) estimates between per 10 units increase of genetically determined metabolites traits (*P* < 1 × 10^-5^) and prescription opioid use (POU).**

Black dots indicated the IVW estimates (raw beta) after leaving a single SNP in turns. Red dots indicated the pooled IVW estimate (raw beta). Horizontal lines indicated the range of 95% confidence interval.

**
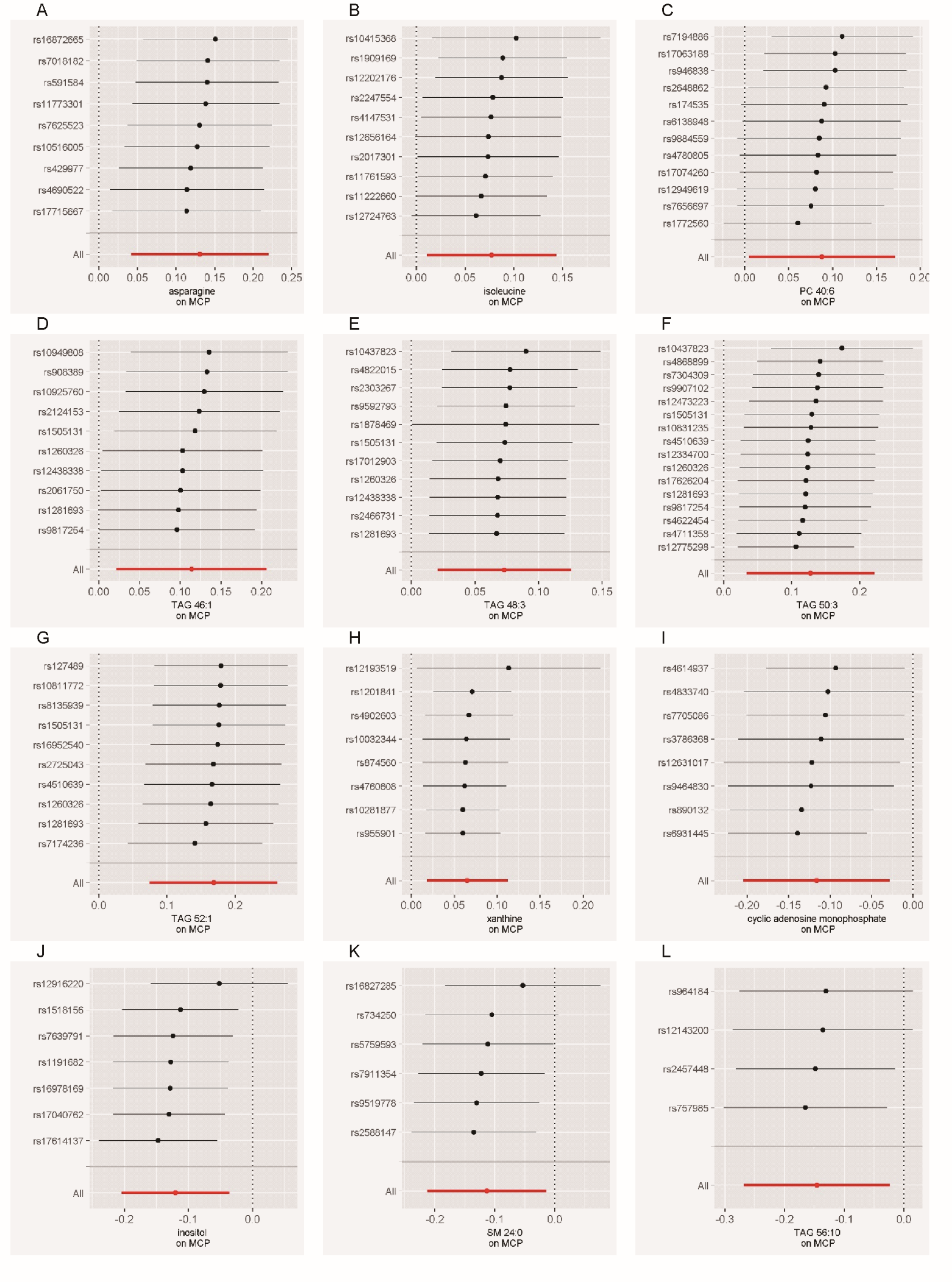
**

**Figure S3. Leave-one-out analysis of inverse-variance weighted (IVW) estimates between per 10 units increase of genetically determined metabolites traits (*P* < 1 × 10^-5^) and multisite chronic pain (MCP).**

Black dots indicated the IVW estimates (raw beta) after leaving a single SNP in turns. Red dots indicated the pooled IVW estimate (raw beta). Horizontal lines indicated the range of 95% confidence interval.

**Additional information 1. Harmonised data.**

A copy of all harmonized data was provided as supplementary data (Shown in the file “Additional information 1. Harmonised data.xlsx”). It contains 6 data sheets including harmonized data for POU on gut microbiota, harmonized data for gut microbiota on POU, harmonized data for MCP on gut microbiota, harmonized data for gut microbiota on MCP, and harmonized data for metabolites on POU. **Abbreviations:** SNP, single nucleotide polymorphism rsid variant identifier; CHR, chromosome; POS, base pair position; EA, effect allele; OA, other allele; PAL, palindromic; F, F-statistics; beta, genetic effects of SNP on exposure/outcome; se, standard error; eaf, effect allele frequency; *P*, *P*-value of the effect estimate. F-statistic for each SNP of exposure was calculated using the formula: F statistics = (beta/se) ^2^ according to Burgess et al. (2016).

**Additional information 2. Results of MR analysis.**

A copy of the results of all MR analyses was provided as supplementary data (Shown in the file “Additional information 2. Results of MR analysis.xlsx”). It contains 6 data sheets including MR estimates for POU on gut microbiota, MR estimates for gut microbiota on POU, MR estimates for MCP on gut microbiota, MR estimates for gut microbiota on MCP, MR estimates for metabolites on POU, and MR estimates for metabolites on MCP. Three methods were used for MR estimates between exposure and outcome, including IVW, MR-egger and Weighted Median, except for those exposures, the number of SNPs eventually included was less than 3. Each data sheet contains the following information, Exposure, Outcome, Method, NSNPs, OR (95%CI) or Beta (95%CI), *P*-value for MR estimates, Egger intercept (*P*) and MRPRESSO global test RSSobs (*P*) for Directional pleiotropy, I^2^ statistics and Q-*statistic* (*P*) for Cochran Q-test, Q'-statistic (P) and Q–Q' (*P*) for Rucker's framework, Steiger *P* for directionality test. **Abbreviations:** POU, prescription opioid use; MCP, multisite chronic pain; IVW, inverse variance weighted; NSNPs, number of single nucleotide polymorphisms; Beta, MR effect estimate; OR, odds ratio; CI, confidence interval; RSSobs, residual sums of squares of observations.
